# *Sec1* regulates intestinal mucosal immunity in a mouse model of inflammatory bowel disease

**DOI:** 10.1186/s12865-023-00578-9

**Published:** 2023-12-08

**Authors:** Jing Cai, Hao Wu, Chenxing Wang, Yujiao Chen, Dingli Zhang, Shiwei Guan, Beilei Fu, Yingli Jin, Cao Qian

**Affiliations:** 1 Department of Gastroenterology, Sir Run Run Shaw Hospital, College of Medicine, Zhejiang University, Hangzhou, 310016 China; 2 Inflammatory Bowel Disease Center, Sir Run Run Shaw Hospital, College of Medicine, Zhejiang University, Hangzhou, 310016 China; 3 Department of Comprehensive Medicine, The Second, Wenzhou Central Hospital Medical Group, Affiliated Hospital of Shanghai University, Affiliated Dingli Clinical Institute of Wenzhou Medical University, Wenzhou, 325000 China; 4 Department of Gastroenterology, The Second Affiliated Hospital, Yuying Children’s Hospital of Wenzhou Medical University, Wenzhou, 325000 Zhejiang China; 5 Department of Hepatobiliary Surgery, Wenzhou Central Hospital, The Dingli Clinical Institute of Wenzhou Medical University, Wenzhou, 325000 Zhejiang P.R. China; 6 Institute of Gastroenterology, Zhejiang University, Hangzhou, 310016 Zhejiang China

**Keywords:** Inflammatory bowel Disease (IBD), *Sec1*, *fut2*, Fucosylation, Death receptors 5 (DR5), Epithelial cell barrier

## Abstract

Inflammatory bowel disease (IBD) is a common immune-mediated condition with its molecular pathogenesis remaining to be fully elucidated. This study aimed to deepen our understanding of the role of *FUT2* in human IBD, by studying a new surrogate gene *Sec1*, a neighboring gene of *Fut2* and *Fut1* that co-encodes the α 1,2 fucosyltransferase in mice. CRISPR/Cas9 was used to prepare *Sec1* knockout (*Sec1*^*−/−*^) mice. IBD was induced in mice using 3% w/v dextran sulphate sodium. Small interfering RNA (siRNA) was employed to silence *Sec1* in murine colon cancer cell lines CT26.WT and CMT93. IBD-related symptoms, colonic immune responses, proliferation and apoptosis of colon epithelial cells were assessed respectively to determine the role of *Sec1* in mouse IBD. Impact of *Sec1* on the expression of death receptor 5 (DR5) and other apoptosis-associated proteins were determined. *Sec1* knockout was found to be associated with deterioration of IBD in mice and elevated immune responses in the colonic mucosa. Silencing *Sec1* in CT26.WT and CMT93 cells led to greater secretion of inflammatory cytokines IL-1β, IL-6 and TNF-α. Cell counting kit 8 (CCK8) assay, flow cytometry and TUNEL detection suggested that *Sec1* expression promoted the proliferation of colon epithelial cells, inhibited cell apoptosis, reduced cell arrest in G0/G1 phase and facilitated repair of inflammatory injury. Over-expression of DR5 and several apoptosis-related effector proteins was noticed in *Sec1*^*−/−*^ mice and *Sec1*-silenced CT26.WT and CMT93 cells, supporting a suppressive role of *Sec1* in cell apoptosis. Our results depicted important regulatory roles of *Sec1* in mouse IBD, further reflecting the importance of *FUT2* in the pathogenesis of human IBD.

**Supplementary Information:**

The online version contains supplementary material available at 10.1186/s12865-023-00578-9.

## Research highlights

*Sec1*, a proxy gene of human *fut2*, plays a protective role against mouse IBD.

*Sec1* negatively modulates the secretion of key intestinal inflammatory factors.

*Sec1* supports cell proliferation and regulates DR5 fucosylation and IEC apoptosis.

**Supplementary Information:**

The online version contains supplementary material available at 10.1186/s12865-023-00578-9.

## Introduction

Inflammatory bowel disease (IBD) is a chronic and non-specific intestinal inflammatory disease with high mortality and morbidity [1, 2]. The etiology of IBD is known to be related to genetic susceptibility, environmental cues, immune imbalance and some other factors [3]. A significant correlation has been established between IBD and colon cancer, with more than 20% of patients diagnosed with IBD further developing colon cancers in the next 30 years [4]. Although a complete cure for IBD is yet to be achieved, advances in immunotherapies that directly target the pathogenesis of IBD have seen a significant improvement in patient outcomes [5].

The pathogenesis of IBD and its underpinning molecular drivers are yet to be fully elucidated. Alterations in fucosylation of glycoproteins in humans, a glycosylation process catalyzed by fucosyltransferases (FUTs), has been linked to several immune disorders including IBD [6]. α2-fucosyltransferases are key enzymes for the fucosylation process and are encoded by *fut1* and *fut2* in humans [7]. Among the 13 currently known human FUTs, FUT2 is the only one forming α-1,3 and α-1,4 fucosidic bonds, allowing syntheses of LEX, LEY and sLEX using its α-1,3- fucosyltransferase function, and LEA, LEB and sLEA with its α-1,4- fucosyltransferase function [8]. sLEX and sLEA work as ligands for cell adhesion molecules E-selectin and P-selectin and play an important role in the recruitment of leukocytes and platelets when tissue injury occurs [9].

Importance of *FUT2* in human IBD has been recently reported by Tang et al. (2021) using *Fut2* knockout (KO) mice and a mouse model of colitis [10]. In mice, *Fut2* was found to be important for the synthesis of lysophosphatidylcholine and the composition and function of gut microbiota; knockout of *Fut2* promoted intestinal inflammation and induced epithelial barrier damage [10]. Decreases in *FUT2* expression and α-1,2-fucosylation were also noticed in the colon of patients with IBD such as ulcerative colitis (UC) or Crohn’s disease (CD) [10]; an increased expression of Lewis A antigen in the crypt epithelium of bowel tissues was also found in patients with deficiency in FUT2 synthesis [10].

The death receptor (DR) is a key pro-apoptotic factor found in many healthy cells [11]. A study conducted on human colorectal cancer cell lines by Zhang et al. (2019) found DR5-related apoptosis heavily relied on the expression of specific fucosyltransferases such as FUT3 and FUT6 [12]. The involvement of N-acetylgalactosyltransferase and fucosyltransferases triggers O-glycosylation of DRs, facilitating the binding of N-acetylgalactose to Ser/Thr residues of DR extracellular polypeptide, and consequentially enhancing the glycosylation of DR4 and DR5 [12]. More recently, Park et al. (2020) found that the O-glycosylation of DRs might increase their membrane stability and hinders endocytosis, ultimately amplifying cell sensitivities to TNF-related apoptosis-inducing ligand (TRAIL)-mediated apoptosis [13]. In addition to the effects on cell apoptosis, DRs may also play an important role in inflammatory regulation and immune homeostasis, contributing to the imbalanced conditions often encountered in IBD [14].

*Fut2* knockout mice lacking of the expression of α [1, 2]fucosylated glycans are now commercially available. It is, however, reasonable to speculate that *fut2* in mice may not fully represent its counterpart in humans as another neighboring gene, *Sec1*, was also found to be highly homologous to human *FUT2*. *Fut1/Fut2/Sec1* triple knockout mice with a complete deficiency of α [1, 2]fucosyltransferase has been recently generated and reported in a preprint; this triple knockout animals showed no deficiency in viability and development, no changes in colon microbiome but had a complete lack of H blood group antigen [15]. Chen et al. (2019) used a LoxP-neomycin-LoxP cassette to replace a 30 kb region that contains protein-coding regions of both *Fut2* and *Sec1* [15]. Such a knockout strategy might have neglected the significance of *Fut2* and *Sec1* as individual genes for mouse IBD. Although its homolog *SEC1p* in humans has been widely accepted as a pseudogene, *Sec1* in mice is a protein-encoding gene and can be expressed in various tissues including the digestive system [7, 16], implicating a possible role of this gene in mouse IBD that may also mirror the importance of *FUT2* in human IBD.

This study aimed to evaluate the role of *Sec1* in the pathogenicity of mouse IBD, as a surrogate gene for human *FUT2*, and sought to deepen our understanding of the importance of FUT2 in human IBD.

## Materials and methods

### Construction of *Sec1*^*−/−*^ mice and *Sec1-siRNA* intestinal epithelial cells

C57BL/6 mice used in this study were purchased from Cyagen Biosciences (Guangzhou, China) and knockout of *Sec1* (gene ID: 56546) was carried out by Cyagen animal service (Guangzhou, China) using CRISPR/Cas9 gene editing technology that has been published elsewhere [17]. The exon 4 of *Sec1* located on the mouse chromosome 7 was selected as the target site for gRNA target sequences (Table 1). The genotype of *Sec1*^−/−^ mice was verified using polymerase chain reactions (PCR), using the following primers F: 5’-AGGTGACAGAAAGATTCAGAGGTAC-3’, R:5’-GAGTGAGTGTGAGTGTGCTAGAAAC-3’. Undifferentiated colon carcinoma cell lines CT26.WT and CMT93 (Xiamen Immocell Biotechnology Co. Ltd) were used to construct *Sec1-siRNA* intestinal epithelial cells (IEC). The mouse *Sec1* sequence was from the NCBI gene library and used as the target of suppression following the design principle of siRNA (si-m-*Sec1*: GGACACTGTTTACCTGGCT, NC: TTCTCCGAACGTGTCACGT). Cells were maintained in the Dulbecco’s modified Eagle’s medium (DMEM, Gibco, NY) supplemented with 10% fetal calf serum (Excell Bio, Shanghai, China) and 1% penicillin-streptomycin solution. This study was approved by the Experimental Animal Ethics Committee of Wenzhou Medical University (Approval number: wydw-2016-0215). All mice were bred and maintained under specific pathogen free conditions and animal experiments were carried out in accordance with the National Institutes of Health guide for the care and use of Laboratory animals.

**Table 1:** gRNA target sequence

Genes	Sequence
gRNA1	TCACAGTGCTCCAGCGACTCAGG
gRNA2	CCATGACAGTAAACACGTTGGGG

### Mouse model of colitis

Colitis was induced in C57BL/6 wildtype (WT) and *Sec1*^*−/−*^ mice with 3% (w/v) dextran sulphate sodium (DSS) (Colitis grade, 36–50 kDa, MP Biomedicals, Canada) pre-dissolved in drinking water and given ad libitum (days 1–5) as described by others [18]. Body weight, rectal bleeding, diarrhoea and mental status of the animals were monitored daily. As for the mental status, sign of alertness/sleeping were monitored, including being dull or depressed, presenting little response to handling, and being unconscious. Mice were sacrificed by cervical dislocation on day 7 and the peripheral blood and colonic tissues were collected for further analyses.

### Histopathological analysis and fluorescence microscopy

Colonic tissues were fixed with 10% formalin and embedded in paraffin. Sections of 5 μm in thickness were stained with hematoxylin and eosin (H&E) for pathological analysis. For immunofluorescence microscopy, paraffin-embedded tissue sections were incubated with FUT3 antibody (Affinity), DR5 antibody (Invitrogen), CY3 Conjugated AffiniPure Goat Anti-Rabbit IgG (H + L) secondary antibody (Boster Biological Technology), and DAPI (Beyotime Biotechnology, China). Sections were examined using an Olympus digital sight BX53 fluorescence microscope. The apoptosis of colonic epithelial cells was assessed using TUNEL BrightRed Apoptosis Detection Kit (A113-03, Vazyme), following the manufacturer’s instruction.

### Flow cytometry

The numbers of Th17 and Treg cells in the spleen of WT and *Sec1*^*−/−*^ mice with induced colitis were analyzed by flow cytometry. The spleen lymphocytes were isolated using a monocyte separator (LTS1092PK, Tianjin Haoyang Biological Manufacture, China) and resuspended in phosphate buffered saline (PBS). Spleen lymphocytes were counted and stimulated using PMA/Ionomycin (Yeasen, Shanghai, China) for 1 h, followed by harvesting, fixation using IC fixation buffer (eBioscience, USA) and permeabilization using 1× permeabilization buffer (eBioscience, USA). Cells were then incubated with the following antibodies for 30 min on ice: CD4 antibody (eBioscience, USA), CD25 antibody (eBioscience, USA), Foxp3 antibody (eBioscience, USA), IL-17 A antibody (eBioscience, USA), and examined using a flow cytometer (CytoFLEX, Beckman). The numbers of CD4/IL-17 A/Th17 and CD4/CD25/FoxP3/T reg cells were calculated using CtyExpert 2.3.

### Apoptosis and cell cycle analysis

CT26.WT or CMT93 cells were seeded in 6-well plates, at a density of approximately 2 × 10^5^ cells per well. After transfection with SiRNA using the lipofectamineTM 2000 transfection reagent (Invitrogen, USA) for 24 h, cells were harvested and washed with PBS, resuspended in 5 µL AnnexinV-APC/7-AAD and incubated in the dark for 20 min. For cell cycle analysis, SiRNA-treated CT26.WT and CMT93 cells were resuspended in 500 µL propidium iodide (PI) solution for 30 min. Cell apoptosis and cycle were analyzed using the CytoFLEX flow cytometer (Beckman). For cell viability assays, CT26.WT and CMT93 cells were seeded in 6-well plates at a density of 5 × 10^3^ cells per well. After receiving treatments for 24 h, cell viability was assessed using a Cell Counting Kit (CCK-8; E-CK-A362, Elabscience) per the manufacturer’s instructions.

### Western blot

Proteins were extracted from colonic tissues and cell lines using RIPA Lysis Buffer (Beyotime Biotechnology, Shanghai, China) and PMSF non-specific protease inhibitor solution (Aladdin, Shanghai, China), following the manufacturers’ instructions. The protein concentrations were determined using the BCA method (Beyotime Biotechnology, Shanghai, China). Protein extracts were separated by sodium dodecyl sulfate polyacrylamide gel electrophoresis (SDS-PAGE) gels and transferred to Immobilon-P polyvinylidene fluoride (PVDF) membranes (Millipore, Burlington, USA). The membranes with transferred proteins were cut prior to antibody hybridization. Membranes were probed with the following primary antibodies: GAPDH antibody (Goodhere Biology, Hangzhou, China), FUT3 antibody (Affinity), DR5 antibody (Invitrogen) and detected using secondary HRP-coupled antibodies (Goodhere Biology, Hangzhou, China). This experiment was carried out in three biological repeats. Details of how western blot images were prepared and presented can be found in Supplementary file 2.

### Quantitative RT-PCR (q RT-PCR)

Total RNA was extracted with Trizol and reverse transcribed into cDNA with ReverTra Ace qPCR RT Kit (Toyobo, Japan). qPCR was carried out using 2×SYBR Green qPCR Mix (With ROX) (Sparkjade, China) and a fluorescence quantitative PCR instrument (Bio-rad, USA). The relative gene expression was calculated using the 2^−*ΔΔ*CT^ method [19]. The primers used for qPCR were listed in Table 2.

**Table 2:** Sequences of the primers for Real-Time Reverse Transcription Polymerase Chain Reaction (qRT-PCR)

Genes		Sequence
*GAPDH*	Forward:	5′-ATGGGTGTGAACCACGAGA-3′
	Reverse:	5′-CAGGGATGATGTTCTGGGCA-3′
*FUT2*	Forward:	5′-CACCATCAGAGTCAAAGGCC-3′
	Reverse:	5′-TAACGCTCCTCCATCCAGTC-3′
*DR5*	Forward:	5′-TACGGTGTGTCGATGCAAAC-3′
	Reverse:	5′-GCTTATGCCAAGATGCCCAA-3′
*IL-6*	Forward:	5′-AGACTTCCATCCAGTTGCCT-3′
	Reverse:	5′-CATTTCCACGATTTCCCAGAGA-3′
*IL-1β*	Forward:	5′-TCAGGCAGGCAGTATCACTC-3′
	Reverse:	5′-AGCTCATATGGGTCCGACAG-3′
*TNF-α*	Forward:	5′-CGTCAGCCGATTTGCTATCT-3′
	Reverse:	5′-CGGACTCCGCAAAGTCTAAG-3′

### The enzyme-linked immunosorbent assay (ELISA)

The cell supernatants from CT26.WT and CMT93 cells, as well as the serum samples of mice with induced colitis were collected. Cytokine levels, including that for IL-1β, IL-6, and TNF-α were measured using ELISA kits (Elabscience Biotechnology, Wuhan, China) according to the manufacturer’s instructions.

### Statistical analysis

Statistical analysis was performed using IBM SPSS ® Statistics version 22 (IBM Corporation, USA). Normality of data was examined and confirmed with the Kolmogorov-Smirnov test. Variables between groups were analyzed by Student’s *t* test and one-way ANOVA. Results were presented as mean ± standard error of the means or mean ± standard deviation. *p* < 0.05 was considered to be statistically significant.

## Results

### Generation of *Sec1* knockout mice using CRISPR/Cas9

**Fig. 1:**
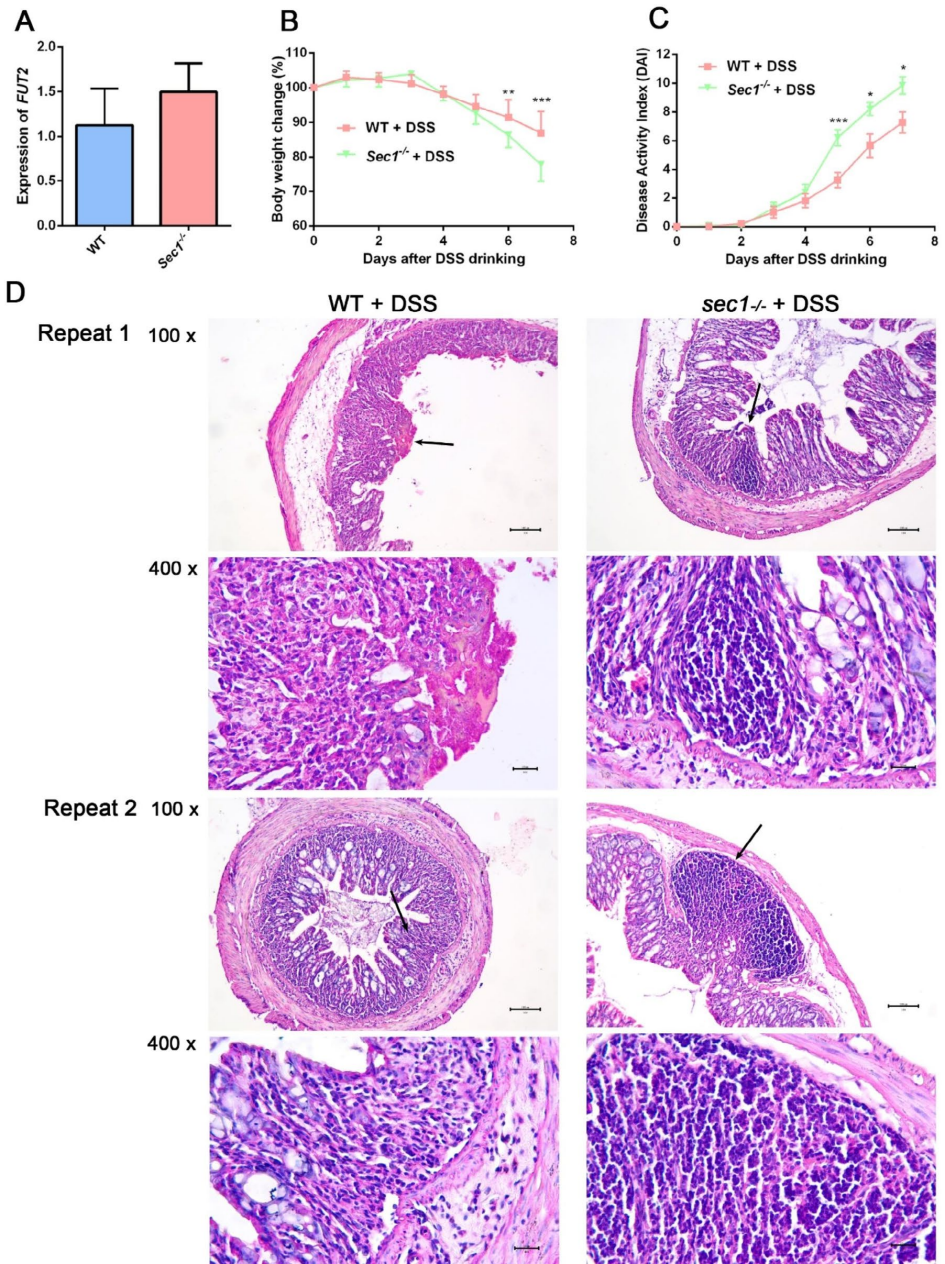
*Sec1* regulated the progression of IBD in mice. (**A**) Gene expression of *Fut2* in WT and the *Sec1*^−/−^ mice were determined by qRT-PCR. (**B**) Changes of body weight in *Sec1*^−/−^ and WT mice after induction of colitis. Data were expressed as the mean ± SEM. n = 15/14. **p < 0.01, ***p < 0.001. (**C**) Disease activity index (DAI) scores of *Sec1*^−/−^ and WT mice after induction of colitis. Data were expressed as the mean ± SEM. n = 15/14. **p < 0.01, ***p < 0.001. (**D**) Histopathological presentation of colonic tissue from *Sec1*^−/−^ and WT mice after induction of colitis. Tissue sections were stained with H&E and imaged at 100 × and 400 ×. Tissues were sampled from the distal colon of mice with DSS-induced colitis. Black arrows indicated areas that were imaged at a higher magnification (400 x). Scale bars were 100 μm (for 100 x) and 20 μm (for 400 x) respectively

Screening PCR confirmed that CRISPR/Cas9 successfully knocked out *Sec1*, with *Sec1*^*−/−*^ and WT animals showing alleles of 432 and 1756 bp respectively. No difference was found in the expression of *Fut2*, the neighboring gene co-encoding the galactoside 2-L-fucosyltransferase with *Sec1* in mice, between colon tissues from the *Sec1*^*−/−*^ and WT animals (Fig. 1A, p > 0.05). Such a finding supported that *Sec1*, was solely responsible for experimental changes in mouse IBD found in this study, not via the effect of mouse *Fut2.*

### *Sec1* played a protective role against mouse IBD

In order to assess the role that *Sec1* plays in the histopathological development of IBD, we induced IBD in *Sec1*^−/−^ and WT C57BL/6 mice. Significantly greater weight loss (*p* < 0.01) was noticed for *Sec1*^−/−^ mice relative to WT mice on day 6 (Fig. 1B); average weight loss reached 20% of the original weight for *Sec1*^−/−^ animals on Day 7. The disease activity index (DAI) was used to evaluate the severity of intestinal inflammation. DAI for both groups increased from day 4, with that of the *Sec1*^−/−^ group becoming significantly higher than the control group on days 5–7 (Fig. 1C, p < 0.001). Histological examination of colonic tissues also revealed typical histopathological changes indicating severe colitis in the *Sec1*^−/−^ group, including severe epithelial ulceration, goblet cell loss, and intraepithelial lymphocyte infiltration, while the wild-type group only showed mild lymphocyte infiltration and mucosal inflammation (Fig. 1D). These findings implicated that *Sec1* played a protective role in the pathogenesis and progression of mouse IBD.

### *Sec1* suppressed in vivo inflammation and inhibited apoptosis of epithelial cells

**Fig. 2:**
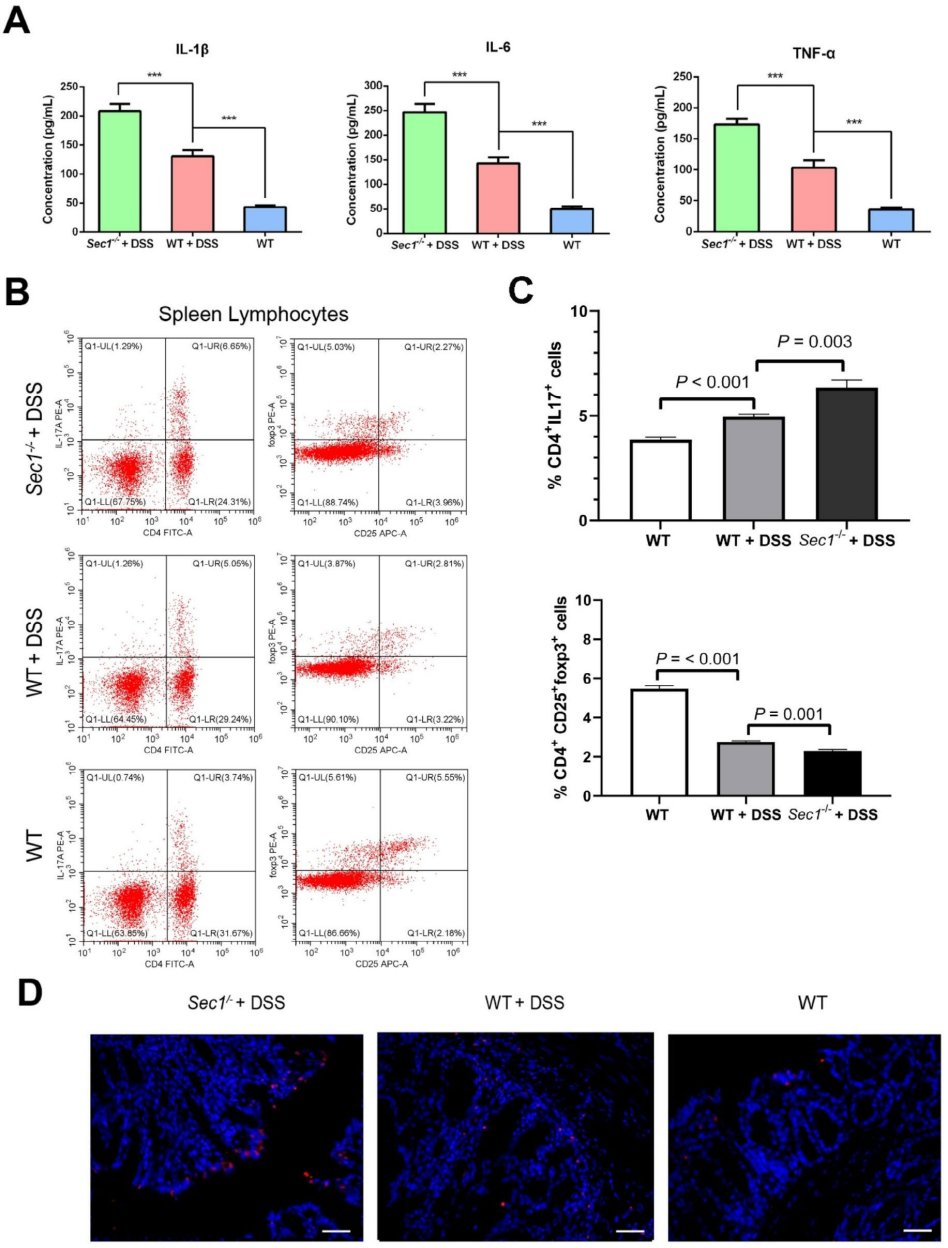
*Sec1* mediates systemic and local inflammation in mice and apoptosis of intestinal epithelial cells. (**A**) ELISA was used to determine the concentrations of IL-1β, IL-6 and TNF-α in the serum of mice. (**B, C**) Qualitative and quantitative analyses of Th17 cells (**B**) and T reg cells (**C**) in spleen lymphocytes of WT, WT + DSS, *Sec1*^−/−^ + DSS mice using flow cytometry. Data were expressed as the mean ± standard deviation. n = 3 biological repeats. (**D**) TUNEL was used to evaluate the apoptosis of colonic epithelial cells in the WT group, WT + DSS group and *Sec1*^−/−^ + DSS group; apoptotic cells showed red fluorescence. The experiment was carried out in three biological repeats. Scale bars = 40 μm

We further evaluated the effect of *Sec1* on the systemic and intestinal immunities in mice. Blood samples were collected from the mouse orbit and the concentrations of several pro-inflammatory factors in the serum were determined. Significantly higher levels of IL-1β (*p* < 0.001), IL-6 (*p* < 0.001), and TNF-α *(p* < 0.001) were found for *Sec1*^−/−^ mice with DSS-induced IBD in comparison to WT animals with same conditions (Fig. 2A), confirming the suppressive role of Sec1 on systemic immune responses. We also isolated lymphocytes from the spleen to assess the T-helper 17 (Th17) T-regulatory (T reg) cell balance. *Sec1*^−/−^ mice with DSS-induced colitis had higher percentage of Th17 (Fig. 2B) and lower percentage of T reg cells than WT animals (Fig. 2B & C), supporting a protective role of Sec1 against mouse intestinal immunity. Cell apoptosis in the intestinal tract of the *Sec1*^−/−^ and WT mice was further determined using TUNEL. Exposure to DSS substantially increased the number of apoptotic cells in the mouse intestine, and knockout of *Sec1* promoted the apoptosis of inflammatory intestine (Fig. 2D).

### Expression of *Sec1* was associated with lowered inflammation, increased cell proliferation and reduced apoptosis in cell cultures

**Fig. 3:**
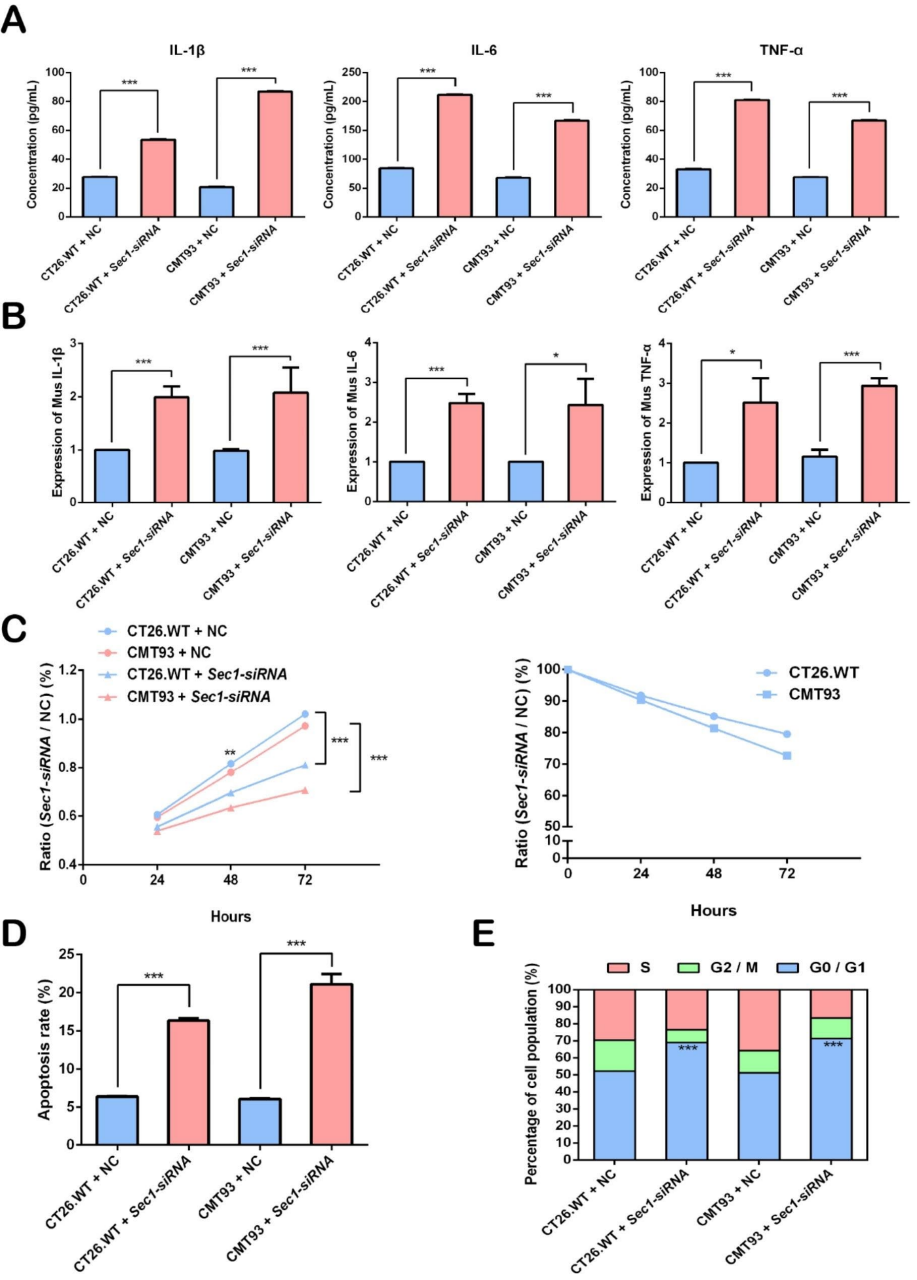
*Sec1* was associated with the down-regulation of inflammatory cytokines, the promotion of epithelial cell proliferation and the inhibition of apoptosis. (**A**) IL-1β, IL-6, TNF-α concentrations in the supernatants of CT26.WT, CMT93, CT26.WT *Sec1-siRNA* and CMT93 *Sec1-siRNA* cells. (**B**) RT-qPCR was used to determine the expression of genes encoding IL-1β, IL-6 and TNF-α. Data were expressed as the mean ± SEM. n = 3. *p < 0.05, ***p < 0.001. (**C**) Cell proliferation. Cell growths of CT 26.WT, CMT93, CT26.WT *Sec1-siRNA* and CMT93 *Sec1-siRNA* cells at 24 h, 48 and 72 h were determined using a spectrophotometer, and ratios of siRNA treated cells to untreated cells were calculated. NC, untreated negative control. (**D**) Apoptosis of siRNA treated and untreated cells. Representative flow cytometry plots can be found in supplementary Fig. 1. (**E**) Cell cycle of siRNA treated and untreated cells. NC, untreated negative control

Supernatants of CT26.WT, CT26.WT *Sec1-siRNA*, CMT93, and CMT93 *Sec1-siRNA* cells were used for comparative analyses. ELISA showed that the concentrations of IL-1β, IL-6 and TNF-α secreted from CMT93 cells after *Sec1* silencing were 4.18 times, 2.46 times and 2.42 times higher than that from untreated control cells (*p* < 0.001, Fig. 3A), while the changes of concentration for IL-1β, IL-6 and TNF-α in CT26.WT cells were 1.92 times, 2.52 times and 2.45 times respectively (*p* < 0.001, Fig. 3A). In agreement with ELISA results, RT q-PCR suggested significantly increased expression of genes encoding inflammatory indicators in *Sec1*-silenced cells relative to that in untreated control cells, including that for IL-1β (CT26.WT: *p* < 0.001, CMT93: *p* < 0.001), IL-6 (CT26.WT: *p* < 0.001, CMT93: *p* < 0.05), TNF-α (CT26.WT: *p* < 0.05, CMT93: *p* < 0.001) (Fig. 3B).

Regenerative healing of the intestinal epithelium is a self-defensive mechanism of the host against IBD [20]. We also examined the protective effect of *Sec1* on intestinal mucosal barrier using CT26.WT and CMT93 and their *Sec1-siRNA* counterparts. CCK-8 colorimetric assays suggested that the proliferation of *Sec1-siRNA* cells at 48 and 72 h were significantly lower than that of the control cells. The proliferation rate of CT26.WT and CMT93 *Sec1-siRNA* decreased by 20.5% and 27.3% respectively when compared with the control group (Fig. 3C).

We further examined the proportion of early and late apoptotic cells in these cell populations using flow cytometry. The apoptosis rates of *Sec1-siRNA* CT26.WT cells and CMT93 cells were found to be 2.55 times and 3.47 times higher than that of the control groups respectively (*p* < 0.001, Fig. 3D and Fig. S1). Flow cytometry also showed that the proportions of CT26.WT *Sec1-siRNA* cells and CMT93 *Sec1-siRNA* cells staying in the G0/G1 phase were significantly higher than that of the control groups (*p* < 0.001, Fig. 3E).

### *Sec1* regulated apoptosis by modulating fucosylation of DR5

**Fig. 4:**
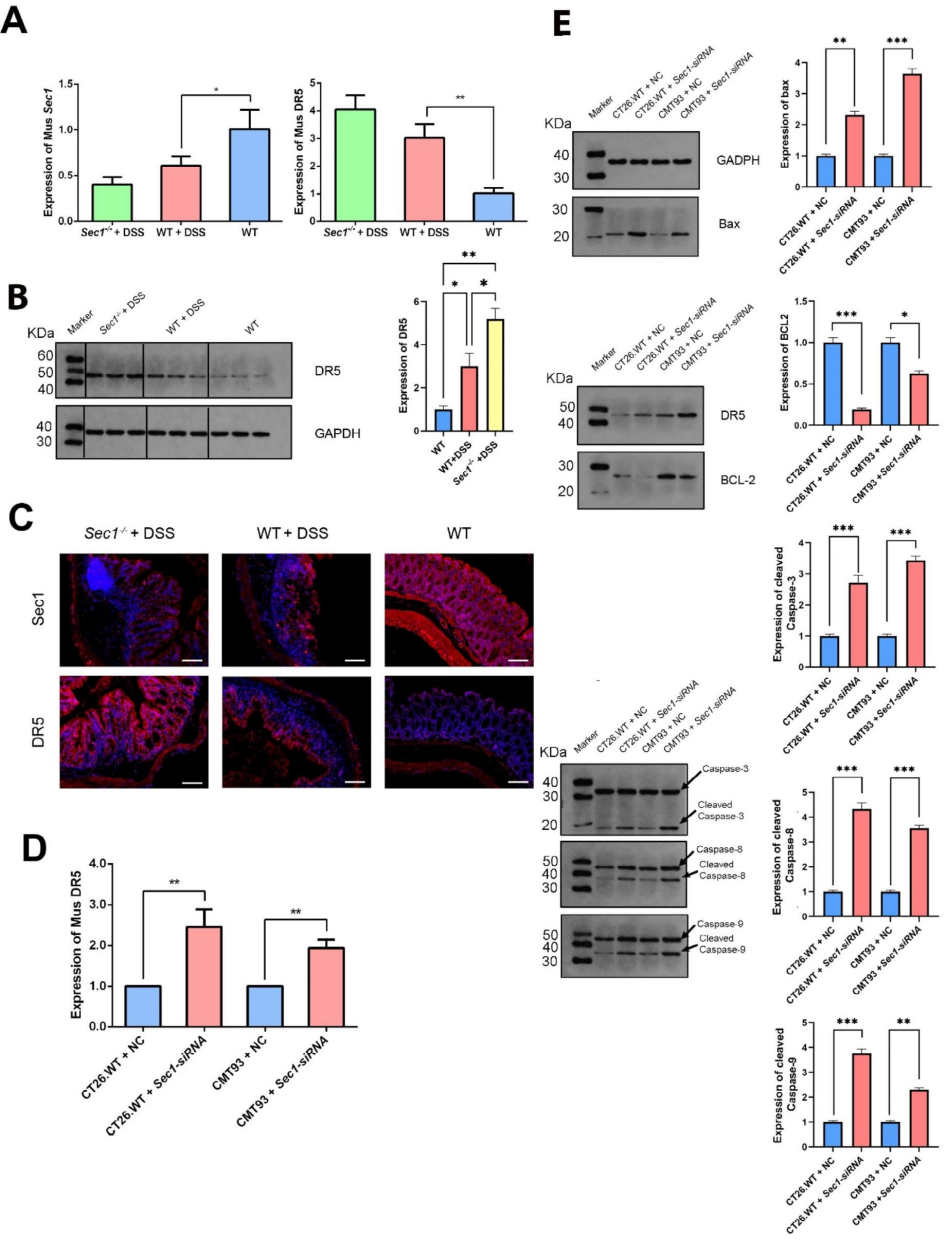
(**A**) *Dr5* gene expression in colonic epithelial cells from WT, WT + DSS, *Sec1*-/- + DSS mice were assessed by QT-qPCR. (**B**) The syntheses of DR5 protein in colonic epithelial cells of three groups were confirmed by western-blot assays. (**C**) Detection of Sec1 and DR5 proteins in mouse colon epithelial cells using immunofluorescence assays. Scale bars = 100 μm. (**D**) *Dr5* gene expression in CT26.WT, CMT93, CT26.WT *Sec1*-siRNA, CMT93 *Sec1*-siRNA cells were determined by QT-qPCR. (**E**) Expression of DR5, and apoptosis-related proteins cleaved Caspase-3, Caspase-8, Caspase-9, Bax and BCL-2 in different groups were determined by western-blot assays. Data were expressed as the mean ± SEM. n = 3. *p < 0.05, **p < 0.01, ***p < 0.001. To be noted, membranes with transferred proteins were cut prior to antibody hybridization

We further investigated how *Sec1* regulated apoptosis, focusing on the fucosylation of the death receptor DR5. Expression of the DR5-encoding gene in WT mice with induced colitis was 2.93 times of that in healthy WT mice (*p* < 0.01, Fig. 4A). Higher expression of *Dr5* was observed in *Sec1*^*−/−*^ mice with induced colitis relative to WT animals receiving the same treatment, at a level approaching the borderline of significance (Fig. 4A, p = 0.074), suggesting that expression of *Dr5* was negatively regulated by the presence of *Sec1*. Western blot assay also showed a significantly higher expression of DR5 in *Sec1*^*−/−*^ mice treated with DSS than that of the colitis or healthy WT mice (Fig. 4B). This was further supported by our immunofluorescent analysis of mouse colon tissues, with DSS-treated *Sec1*^*−/−*^ mice showing stronger fluorescent signals specific for DR5 (Fig. 4C). Taken together, these results suggested that the presence of *Sec1* repressed the expression of death receptor DR5.

We further assessed the expression of DR5 and other key apoptosis proteins in CT26.WT, CMT93 and their *Sec1-siRNA* variants, in order to further identify effector proteins in the DR5-mediated apoptosis signaling pathway. Silencing *Sec1* in CT26.WT and CMT93 increased expression of *Dr5* by 2.46 and 1.94 times respectively (Fig. 4D, p < 0.01). Expressions of key apoptosis proteins, including cleaved Caspase-3, Caspase-8, Caspase-9, Bax and BCL-2 in *Sec1-siRNA* cells were found to be all significantly higher than that in untreated control cells. For instances, the expression of cleaved Caspase-9 in CT26.WT and CMT93 *Sec1-siRNA* cells was 4.56 and 3.35 times higher than that their unsilenced counterparts, and the expression of Bax in CT26.WT and CMT93 *Sec1-siRNA* cells was 6.61 and 5.98 times higher (Fig. 4E).

## Discussion

IBD still remains a severe global health issue with a significant burden of disease [21]. The pathogenesis of IBD and its underlying molecular mechanisms have not been fully clarified, hindering the development of more effective therapies to completely cure this troublesome disease. This study assessed the protective role of *Sec1*, a mouse proxy gene of human *FUT2* against IBD, using *Sec1*^−/−^ mice constructed with CRISPR/Cas9, a mouse model of IBD, and *Sec1-siRNA* intestinal IECs. Our key findings include (1) inactivation of *Sec1* exacerbated symptoms of colitis in a mouse model of IBD, (2) *Sec1* negatively regulated the secretion of key inflammatory factors in the intestinal environment, (3) *Sec1* exerted its protective effect by supporting cell proliferation and regulating DR5 fucosylation and IEC apoptosis.

*FUT2* is known to be involved in the pathogenesis of many diseases in human, including colorectal cancer, pancreatic cancer [22], prostate cancer [23], peptic ulcer [24], atrophic gastritis [25], diabetes [26], and coronary heart disease [27]. Association of *FUT2* with IBD in humans has been studied by several other groups, using *Fut2*^*−/−*^ mice as the proxy [10]. A possible relationship between *FUT2* polymorphism and IBD has been proposed [28], though the mechanism of action of *FUT2* still remains to be clarified. It is questionable whether mouse *Fut2* fully represents human *FUT2* in its importance in the pathogenesis of human IBD. Blast analysis showed *Sec1*, a neighboring gene of *Fut2* in mice, was also highly homologous to human *FUT2*. We reasoned that clarifying the importance of *Sec1* in mouse IBD would be an essential supplement to our current understanding of the role of *FUT2* in human IBD.

We used a mouse model of IBD that was induced by 3% (w/v) DSS. Others have induced colitis in rats with 2,4,6-trinitrobenzenesulfonic acid (TNBS) [29]. Similar to the rat model of TNBS-induced colitis, mouse model of IBD induced by DSS is characterized by increased epithelial damage and abundant production of inflammatory cytokines, similar to severe ulcerative colitis in human [30].We examined Th17 and T reg cells from splenic lymphocytes isolated from IBD mice. Th17 cells mediate inflammatory response while T reg cells mediate immune tolerance; Th17 and T reg cells antagonize each other in biological functions, and the imbalance between these two cells has been considered as the core for the pathogenesis of many diseases including IBD [31]. We found that the presence of *Sec1* promoted the early apoptosis of Th17 cells in mice with induced IBD and tilted the balance to T reg cells. It is thus not surprising that mice in the *Sec1*^*−/−*^ group had a more intense inflammatory response than WT strains.

To further clarify the role of *Sec1* in cell proliferation, apoptosis and cell cycle, we introduced *Sec1-siRNA* to CMT93 and CT26.WT cells. Silencing *Sec1* restored the physical barrier of intestinal epithelial cells by promoting cell proliferation, and negatively regulated cell apoptosis. It is known that apoptosis is involved in embryonic development, cell-mediated immunity and tissue homeostasis [32, 33]. The massive arrest of *Sec1-siRNA* cells in G0/G1 phase implied a lower level of cell proliferation and clonal growth [28]. We deduced that *Fut2* was likely to play a regulatory role in the establishment of epithelial barrier and intestine immunity, two factors that co-mediate the repair of inflammatory injury in the colon.

We also examined the downstream effector of *Sec1* that directly affected cell apoptosis. Several studies have reported that the polymorphism of DR5 increases the risk of Crohn’s disease [34, 35]. Using in vitro and in vivo assays, we were able to identify *Dr5* as an important downstream effector gene of *Sec1* that impacted cell apoptosis [36], possibly via regulating several apoptosis-related proteins, including Caspases, BCL-2 and Bax. The association between these proteins and cell apoptosis have been well established [37, 38]. It is reasonable to believe that the presence of *Sec1*/*Fut2* negatively affects the fucosylation of DR5, thereby inhibiting its excessive role in apoptosis-related pathways.

It has been reported that Gut microbiota in *Fut2*^△IEC^ mice is altered structurally and functionally, promoting generation of LPC which was proved to promote inflammation and damage epithelial barrier [10]. We previously reported that *Fut2* polymorphism might have indirect impact on gut microbiota and intestinal immunity, by regulating the synthesis of Lewis antigen [39]. Intestinal dysbacteriosis selectively activates or inhibits signaling pathways such as aryl hydrocarbon receptor (AhR) and Notch-2, consequentially regulating the proliferation, differentiation and apoptosis of innate immune cells (such as ILCs) and acquired immune cells (such as CD4 + T cells) [40]. A parallel study is currently underway to investigate how *FUT2* is involved in intestinal dysbacteriosis, modulates the intestinal mucosal immune responses and contributes to the pathogenesis of IBD.

In this study, we assessed the protective role of *Sec1* in mouse IBD immediately after the induction of colon damage, following the method by Torretta et al. (2020). Others have used an extended 7-14-day recovery period to evaluate the protective potentials of IL-33 against DSS-induced colitis in mice [41, 42]. The reported effect of *Sec1* in mouse IBD may be more evident or even different provided the assessment was carried out in the recovery period. Another evident limitation of this study is that in vitro carcinoma cell lines were used to represent the colon. CMT93 and CT26.WT cell lines were chosen due to their easy accessibility and the correlation between IBD and colon cancers. Although it has been established that *Fut2* and *Sec1* in mice can be expressed in multiple tissues and have distinct tissue-specific expression patterns, it remains unclear which cell types within these tissues express *Sec1* [7, 16]. Domino et al. (2001) used in situ hybridization and gene-specific probes and found high-level expression of *Fut2* in the luminal uterine epithelium [7]. *Sec1*, however, expressed much more abundantly in thymus and testes/epididymis relative to uterus, small intestine, and colon [7]. Future experimental exploration is needed to determine whether *Sec1* expression is epithelium-specific or also related to other systems such as immune cells [7]. Primary cell lines such as IEC, peritoneal macrophage, or bone marrow derived macrophages, although being technically difficult to obtain, may be used to gain more specific information on the role of *Sec1* in mouse IBD.

## Conclusion

Using *Sec1* in mice as a unique proxy, we verified that *FUT2*, when expressed abnormally, contributed to the pathogenicity of IBD, by modulating human intestinal mucosal inflammation, cell proliferation and apoptosis. *Sec1* may also be used as a biomarker and a new therapeutic target for IBD.

### Electronic supplementary material

Below is the link to the electronic supplementary material.

Supplementary Material 1

Supplementary Material 2

## Data Availability

The datasets analysed and other materials used in this study are available from the corresponding authors on request.
