# Supplementary figures and images for: Sec1 regulates intestinal mucosal immunity in a mouse model of inflammatory bowel disease
Source: BMC Immunol. 2023 Dec 8;24:51. doi: 10.1186/s12865-023-00578-9 (PMC10704666; doi:10.1186/s12865-023-00578-9)

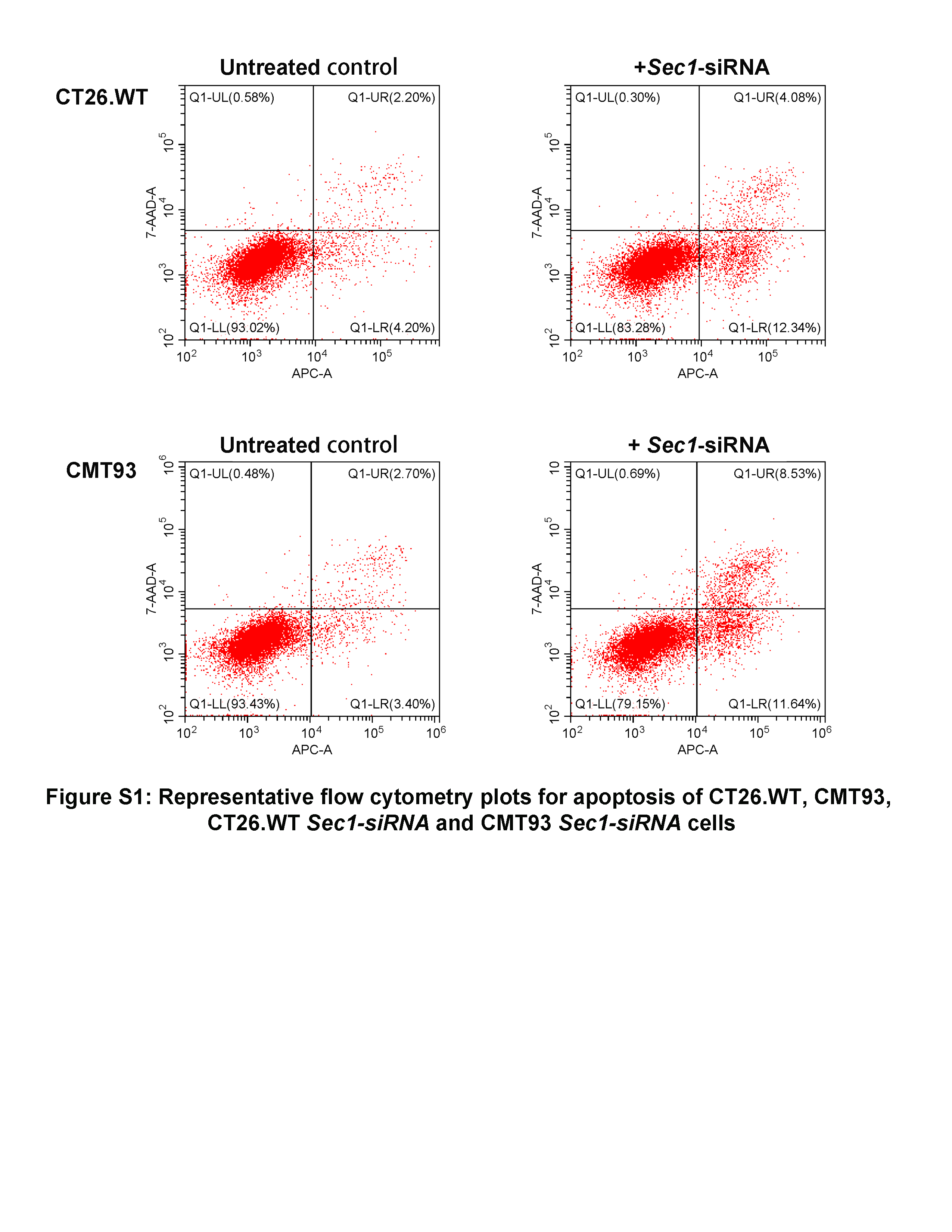

Supplement: Supplementary file 1 — Supplementary Material 1 [file 12865_2023_578_MOESM1_ESM.tif]
