# Supplementary material for: Sec1 regulates intestinal mucosal immunity in a mouse model of inflammatory bowel disease
Source: BMC Immunol. 2023 Dec 8;24:51. doi: 10.1186/s12865-023-00578-9 (PMC10704666; doi:10.1186/s12865-023-00578-9)
Supplement: Supplementary file 2 — Supplementary Material 2 [file 12865_2023_578_MOESM2_ESM.pdf]

Supplementary file 2, original WT blots

To be noted, membranes with transferred proteins were cut/cropped prior to antibody hybridization. No full length gels/blots could be shown.

To clearly show the original bands, the developed WB films were imaged with a multifunction imager (Amersham Imager 680). For bands with weak antibody specificity, dotted lines were used to frame the edge of bands; for bands with strong antibody specificity and a clean background, dotted lines were used to separate different lanes.

Figure 4B: DR5

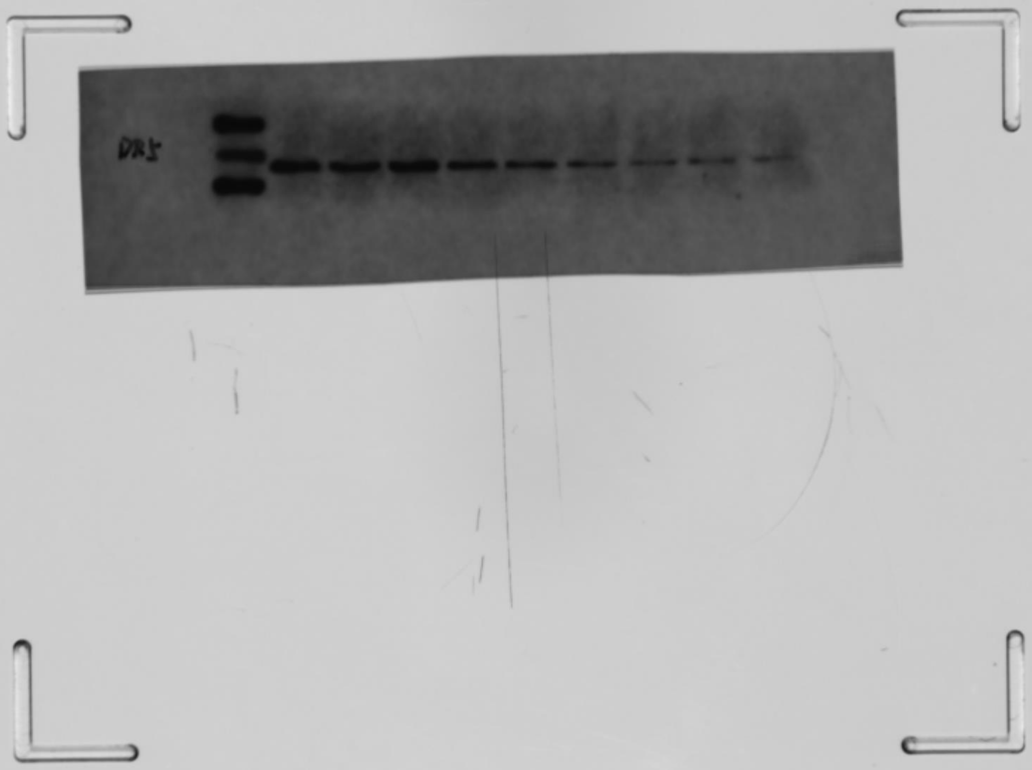

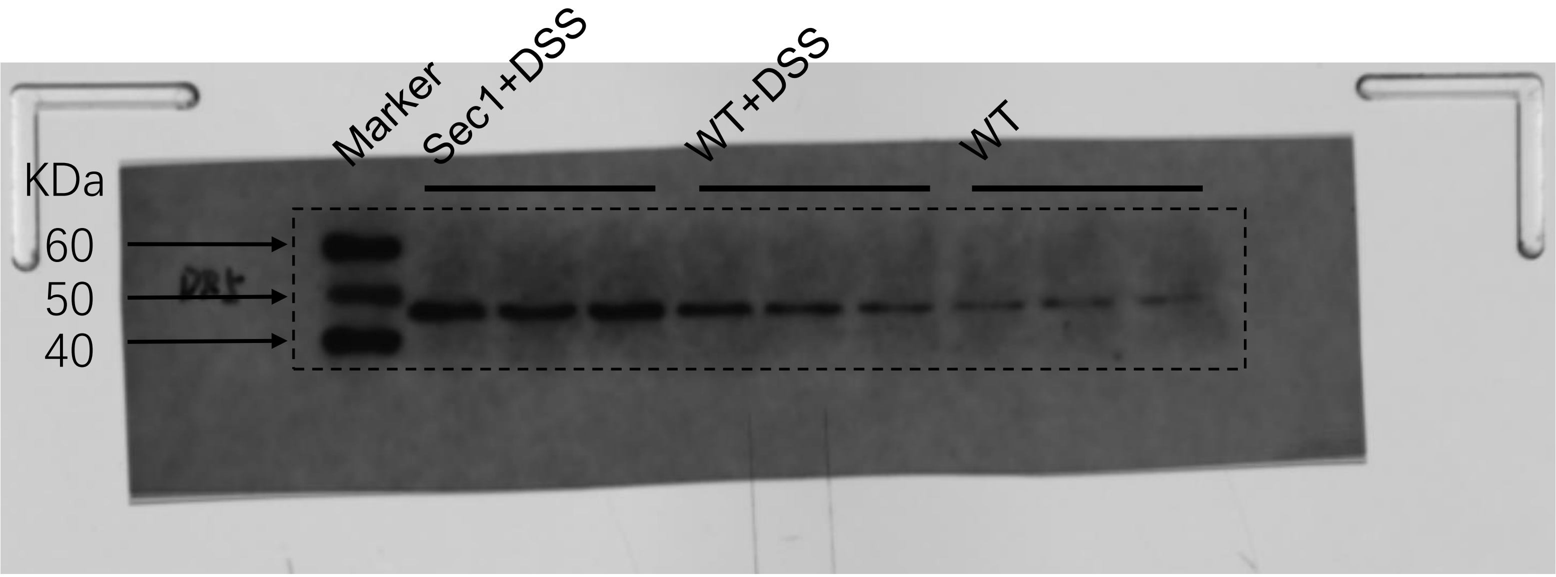

Figure 4B: DR5

Figure 4B: GAPDH

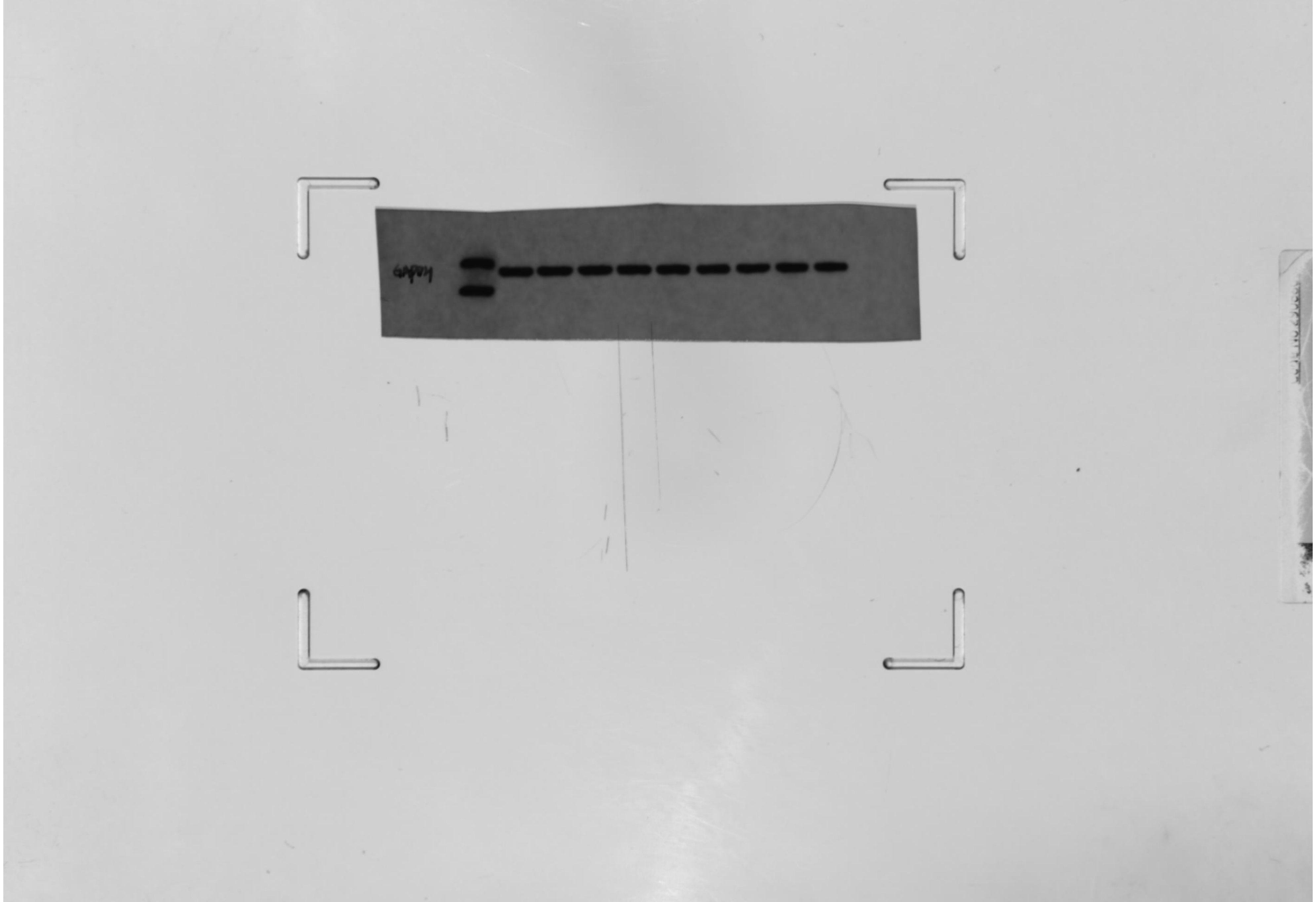

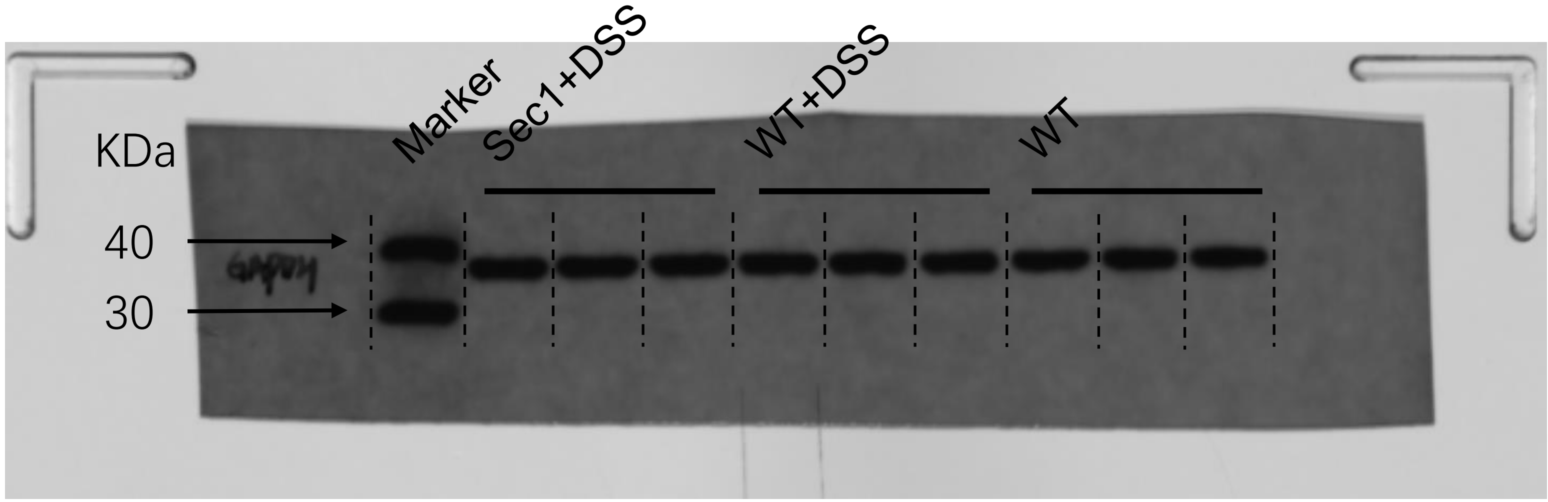

Figure 4B: GAPDH

Figure 4E: GAPDH1

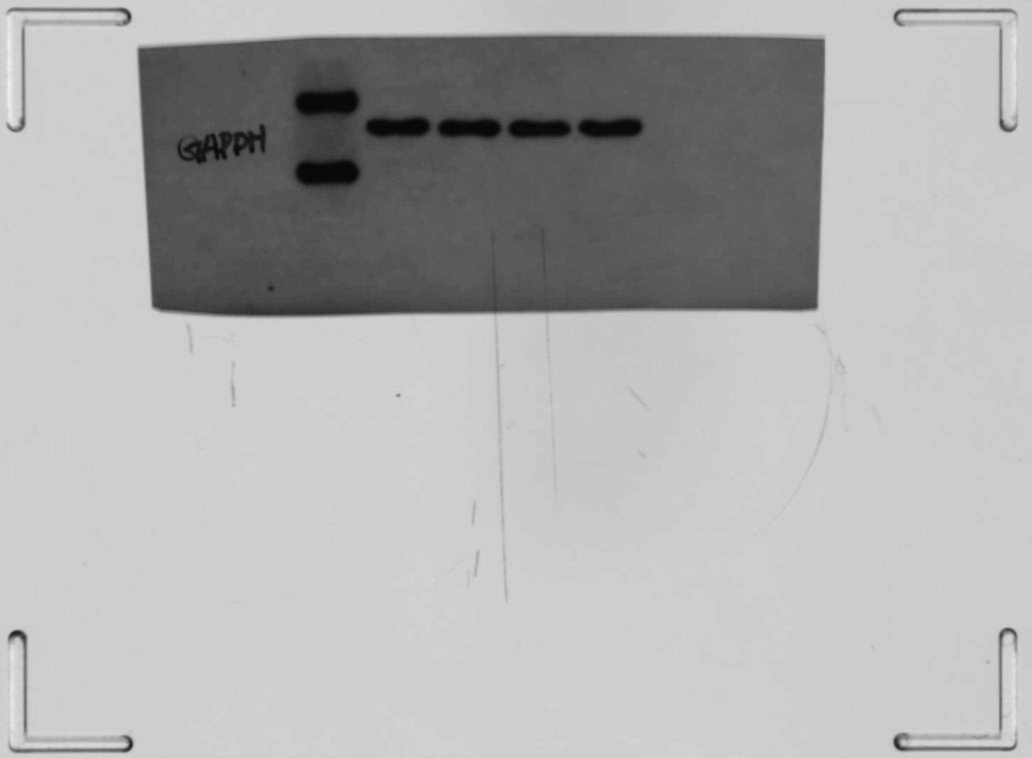

GAPDH2

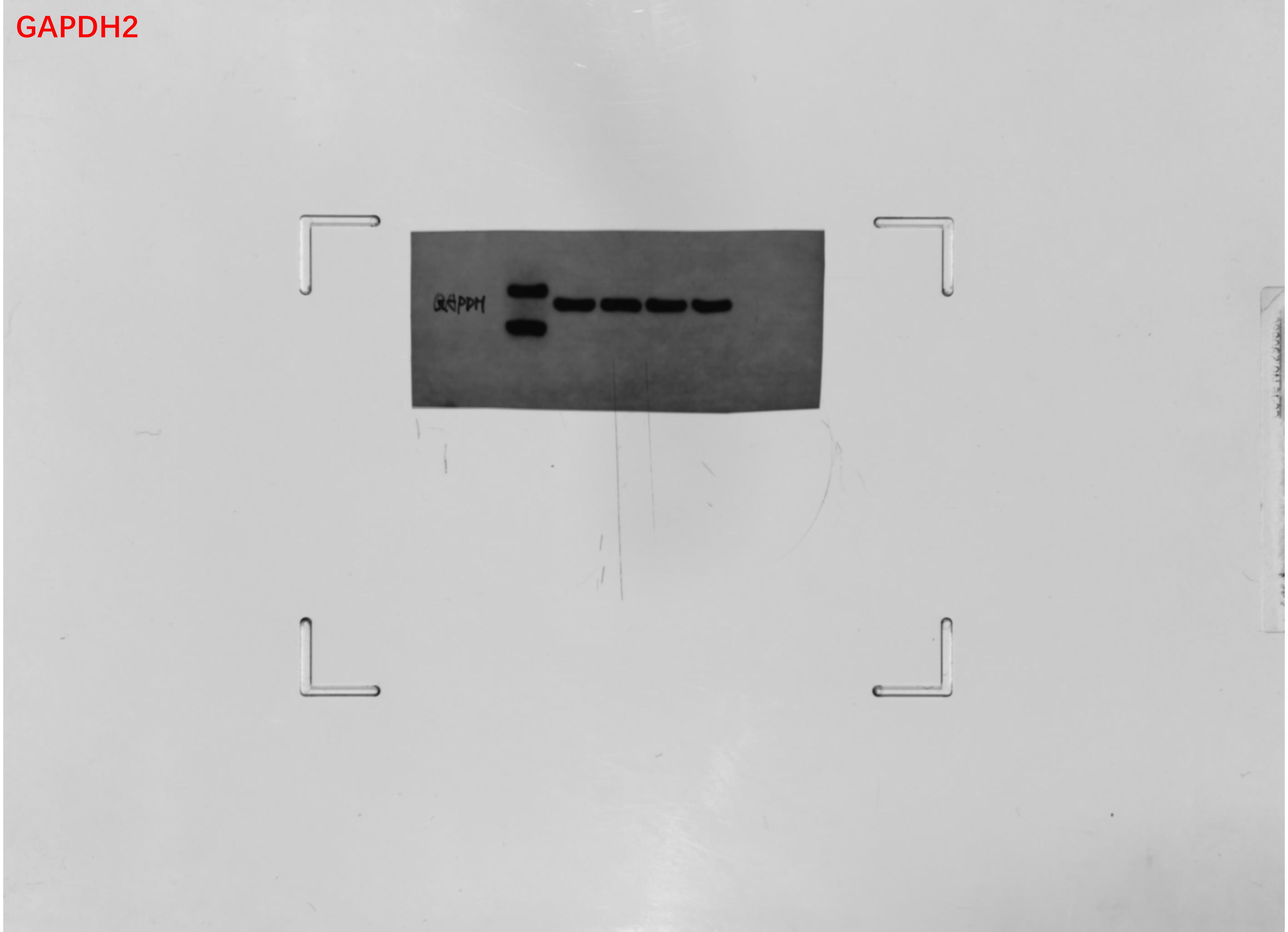

GAPDH3

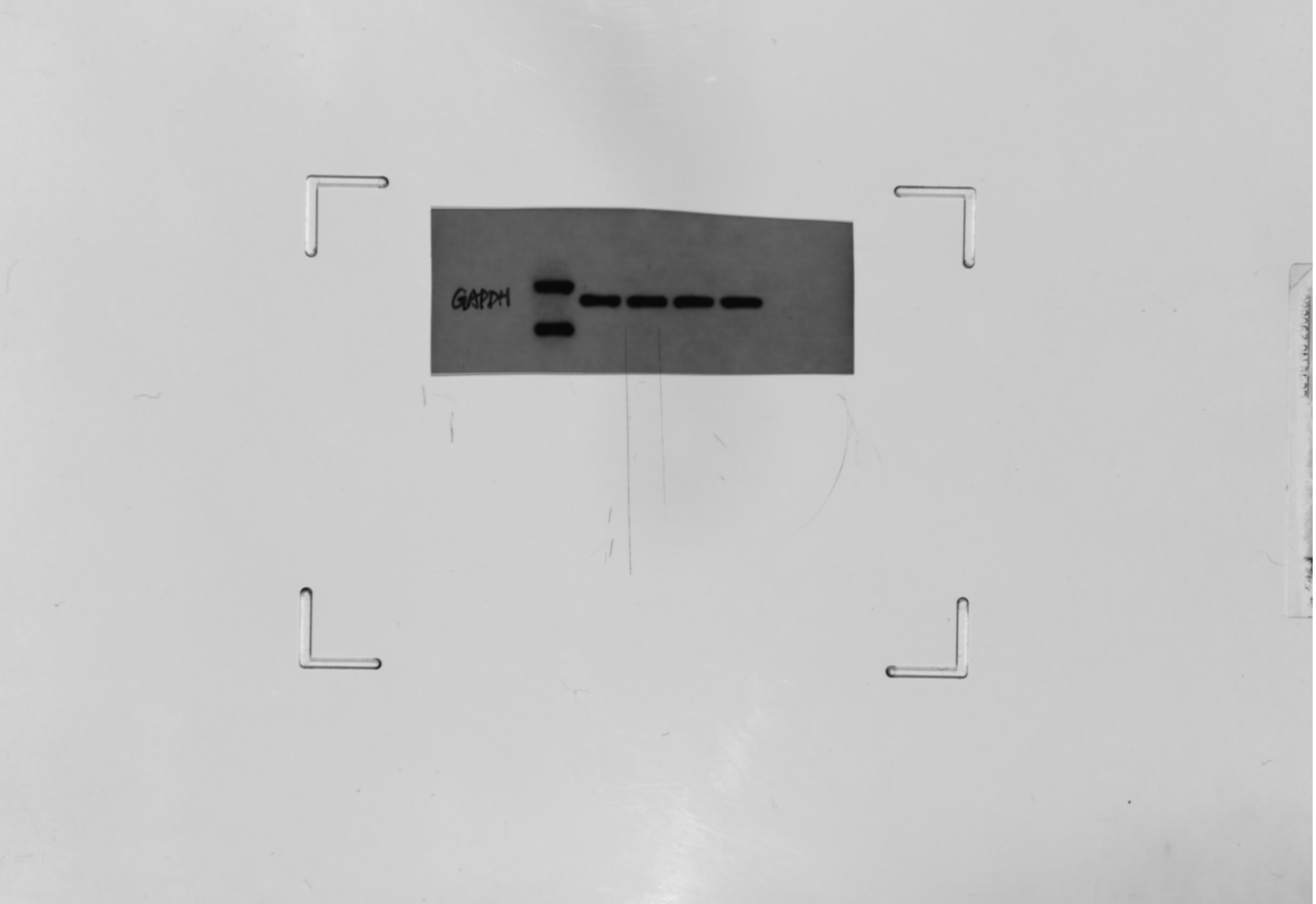

Figure 4E: GAPDH

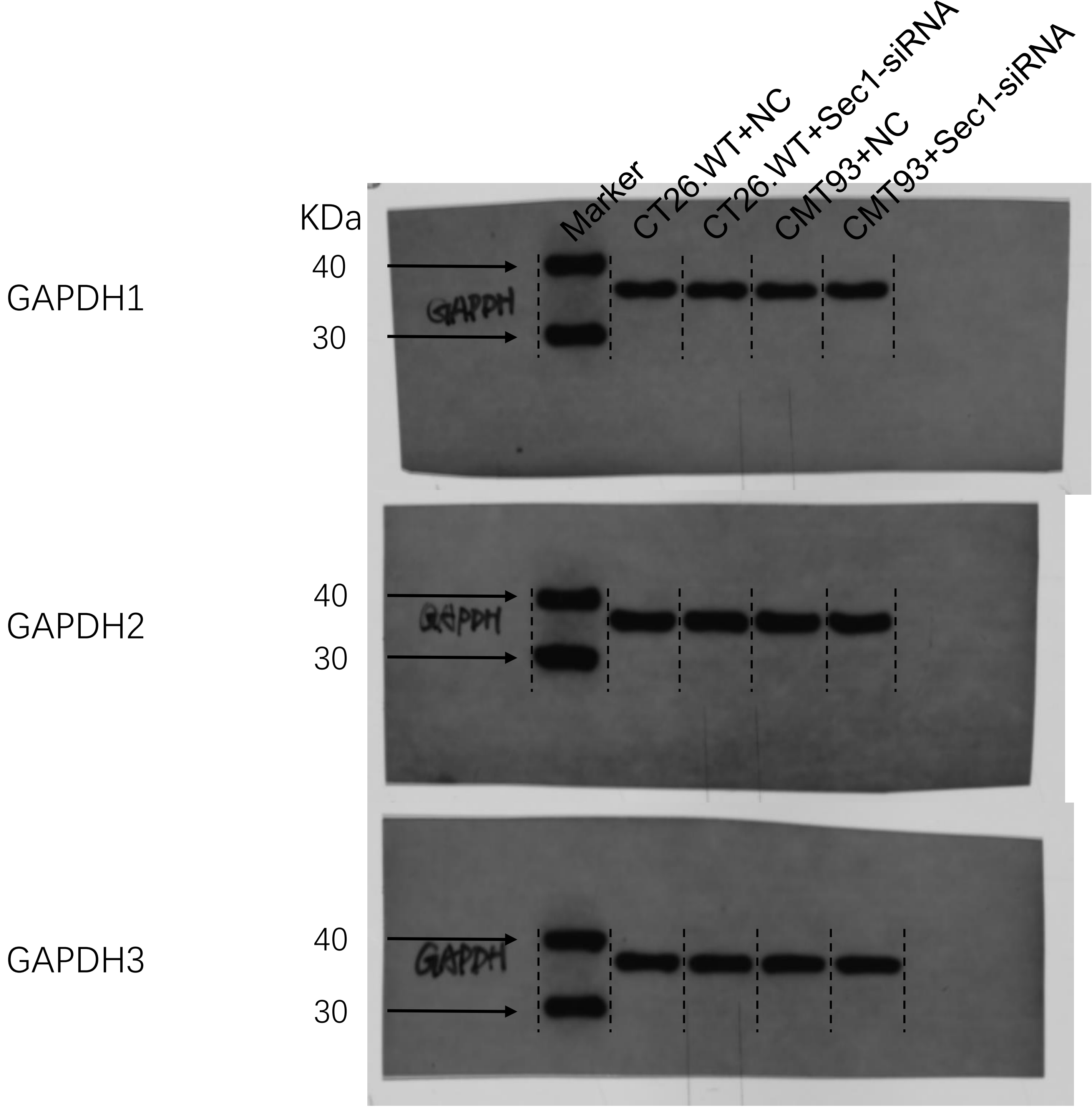

Figure 4E: BAX

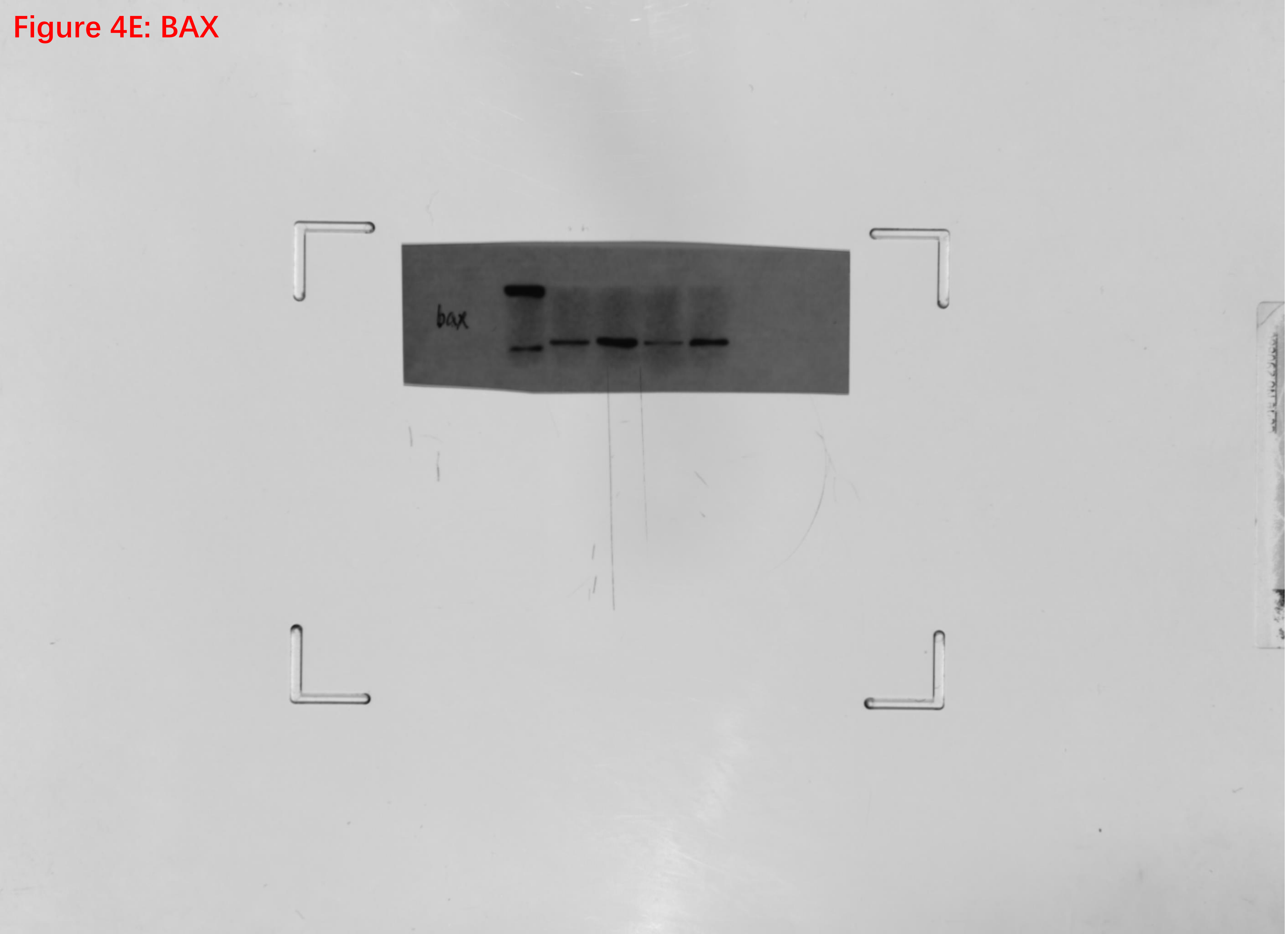

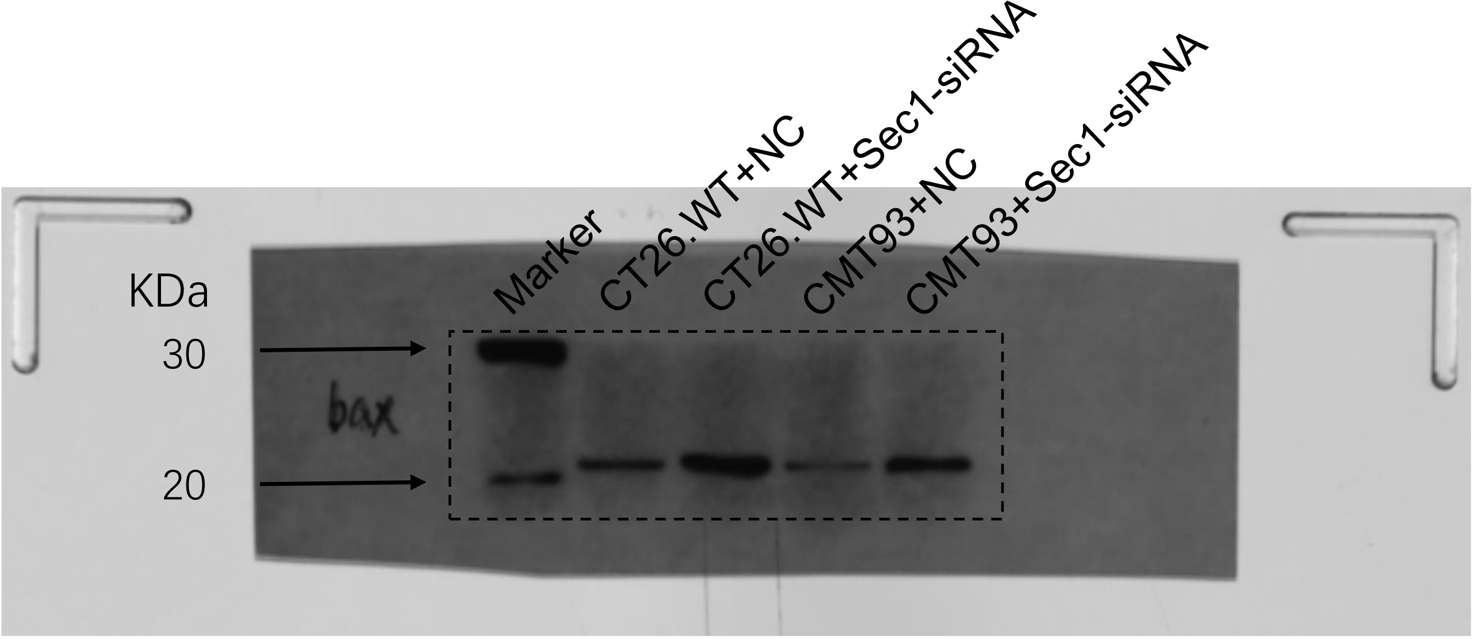

Figure 4E: BAX

Figure 4E: DR5

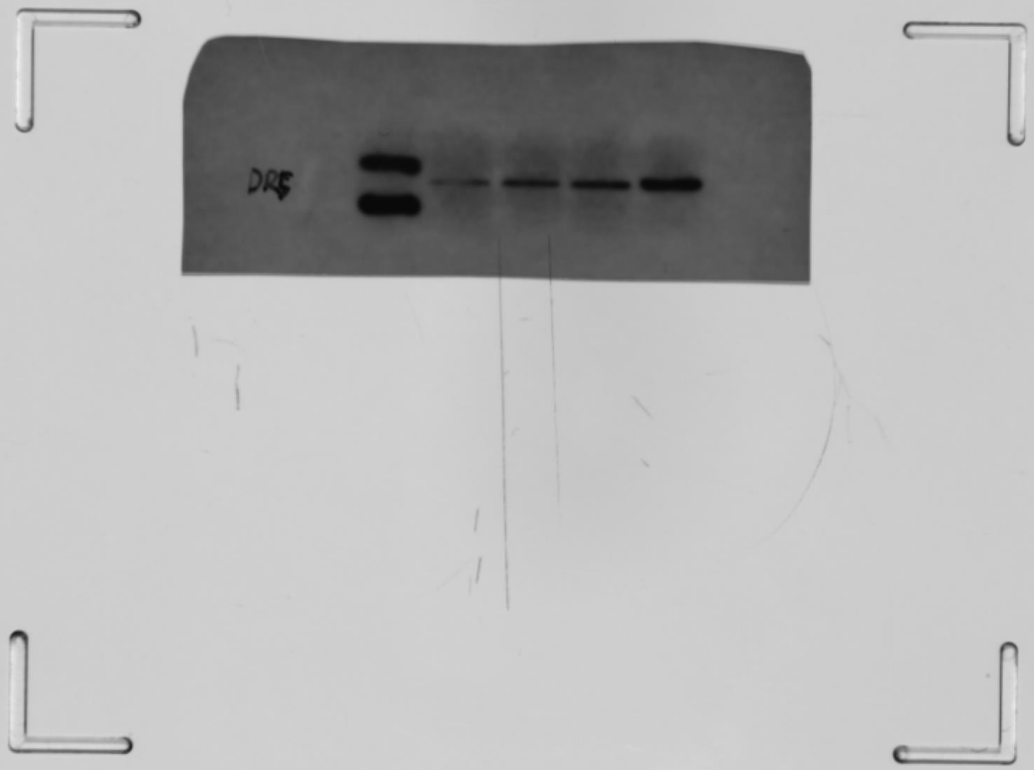

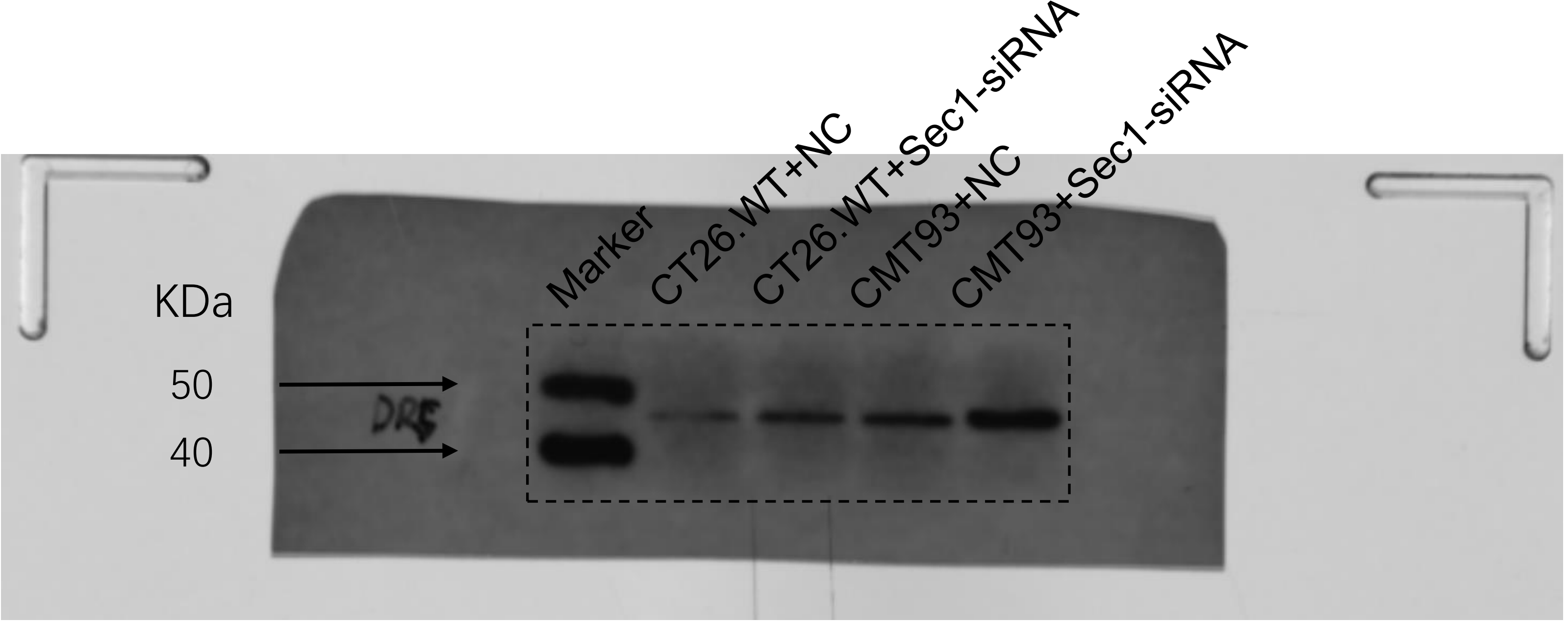

Figure 4E: DR5

Figure 4E: BCL-2

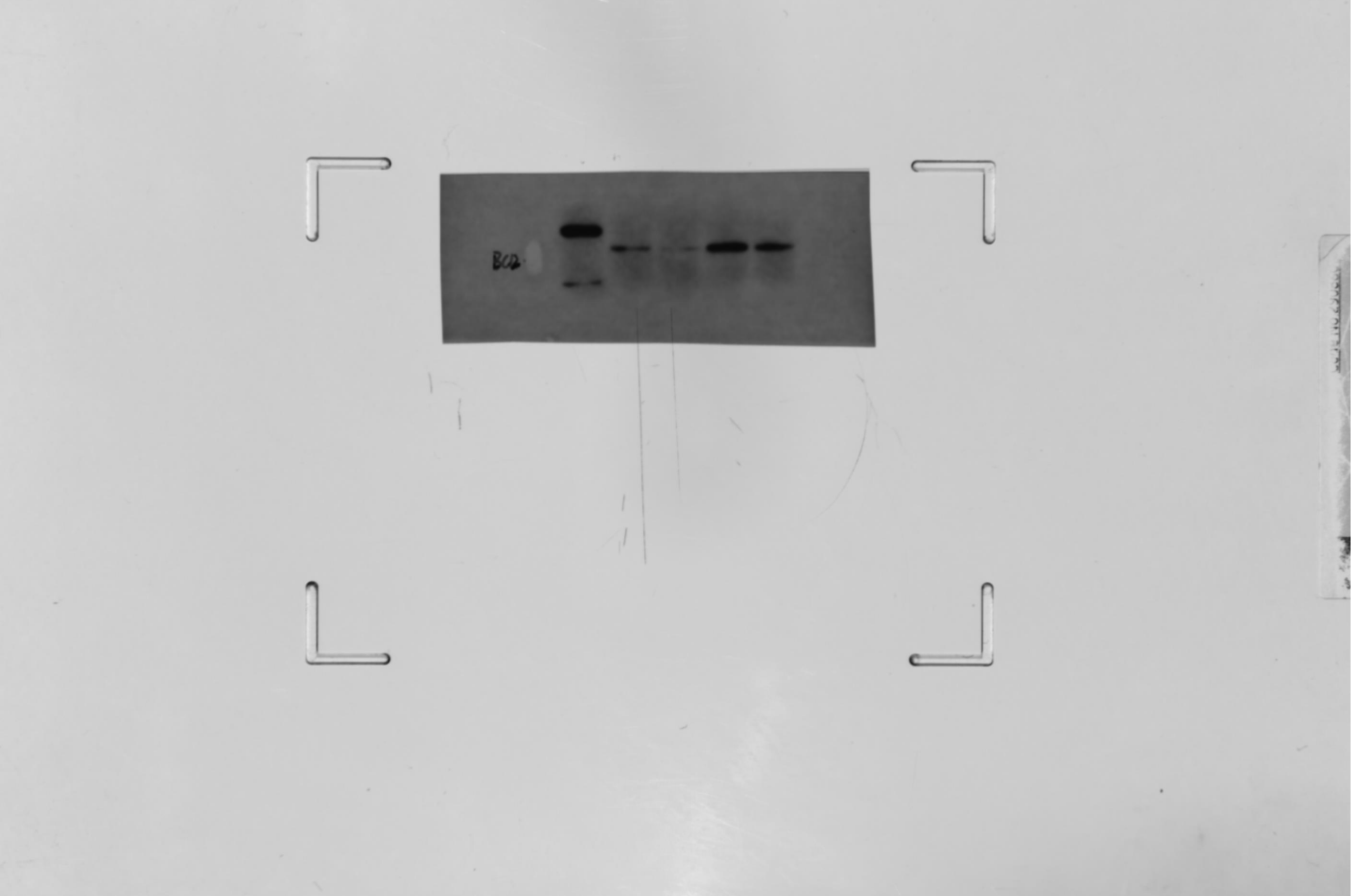

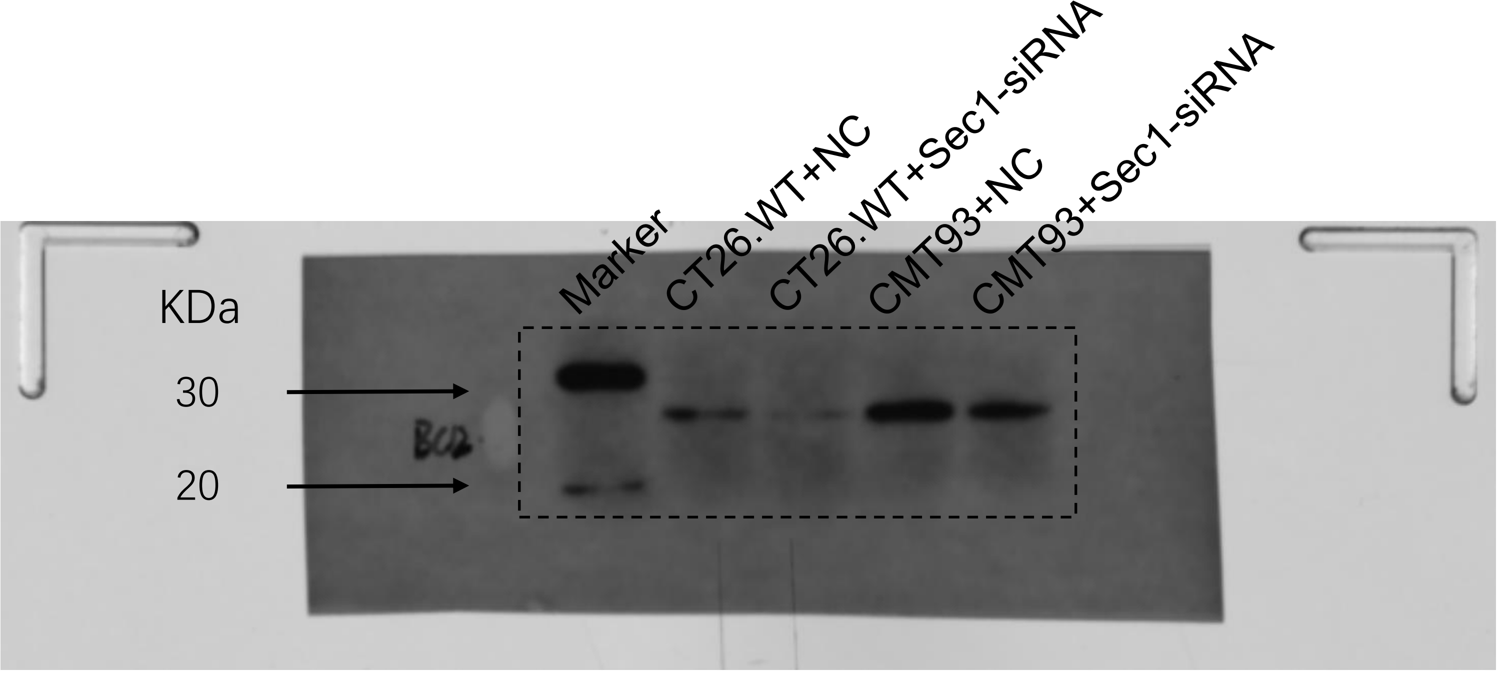

Figure 4E: BCL-2

Figure 4E: CASPASE3

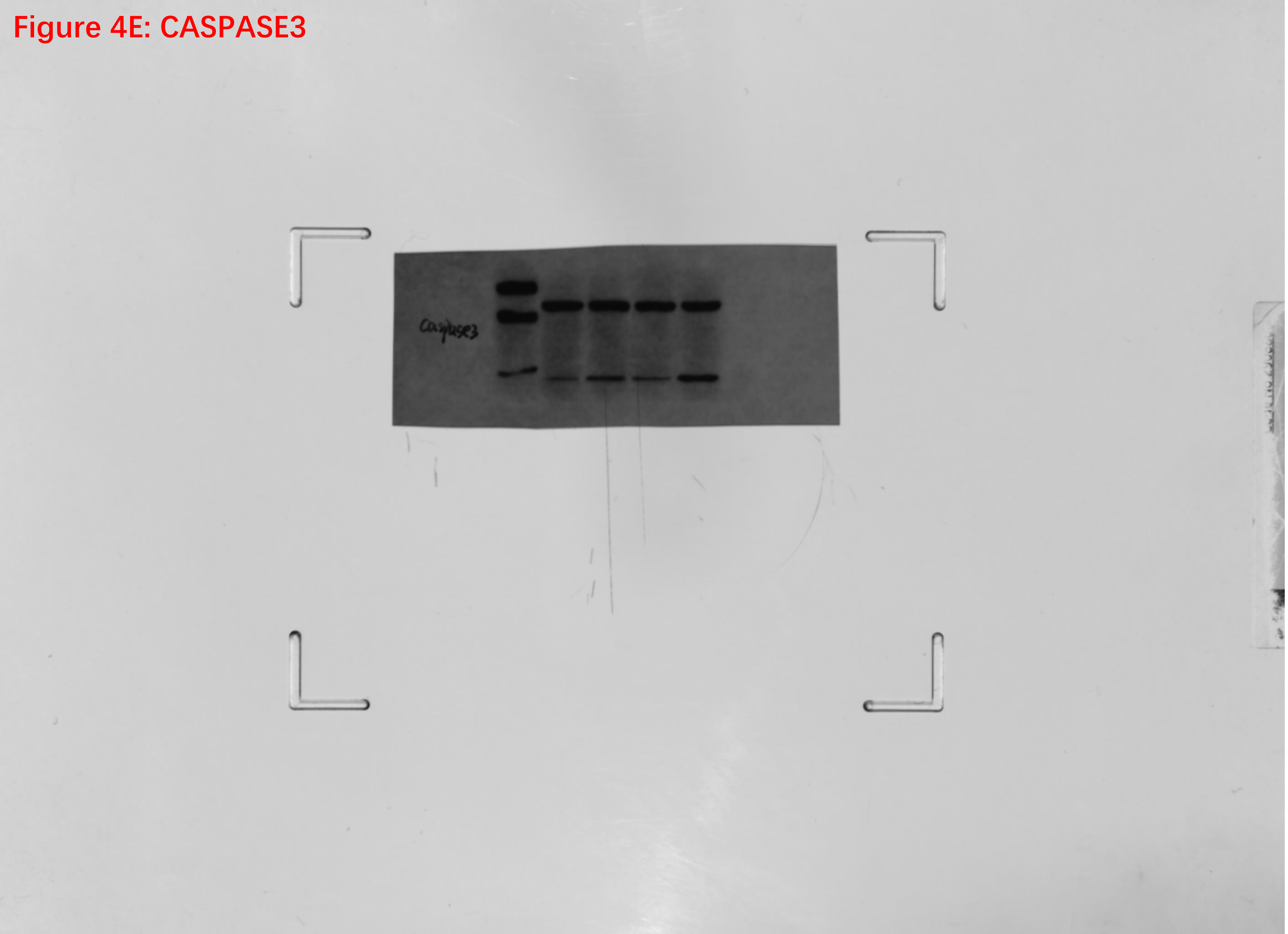

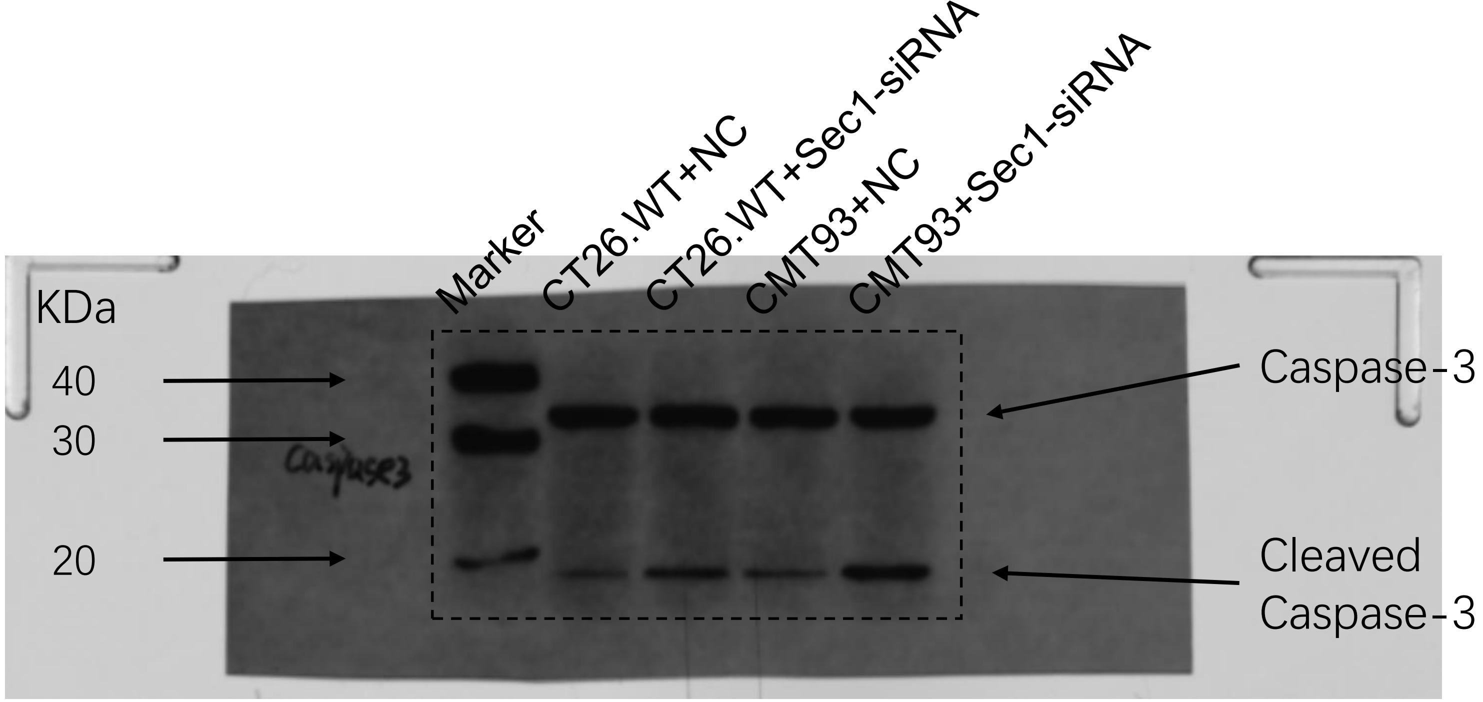

Figure 4E: CASPASE3

Figure 4E: CASPASE8

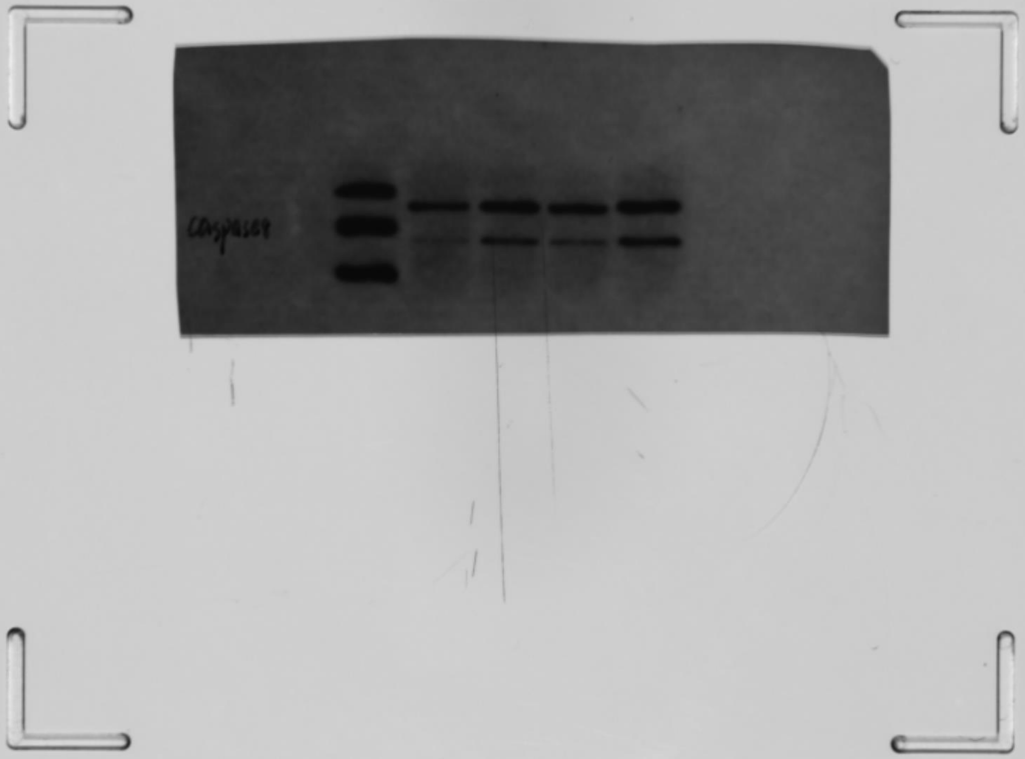

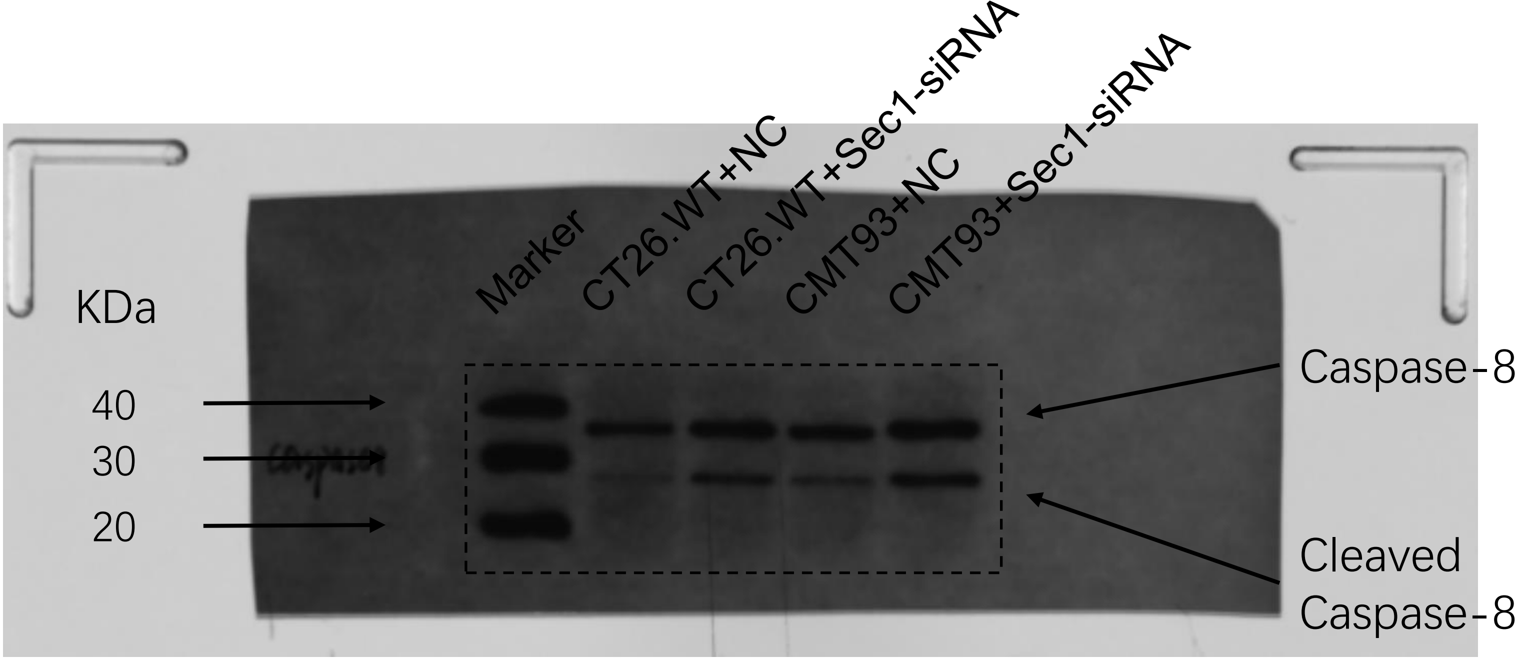

Figure 4E： CASPASE8

Figure 4E: CASPASE9

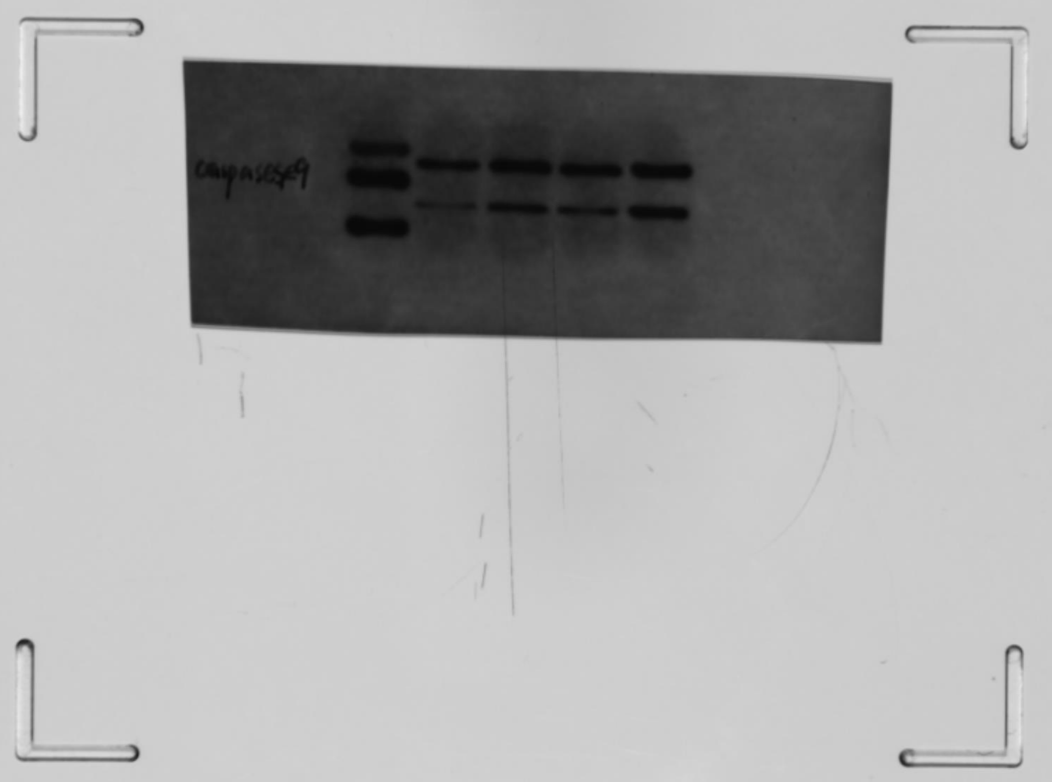

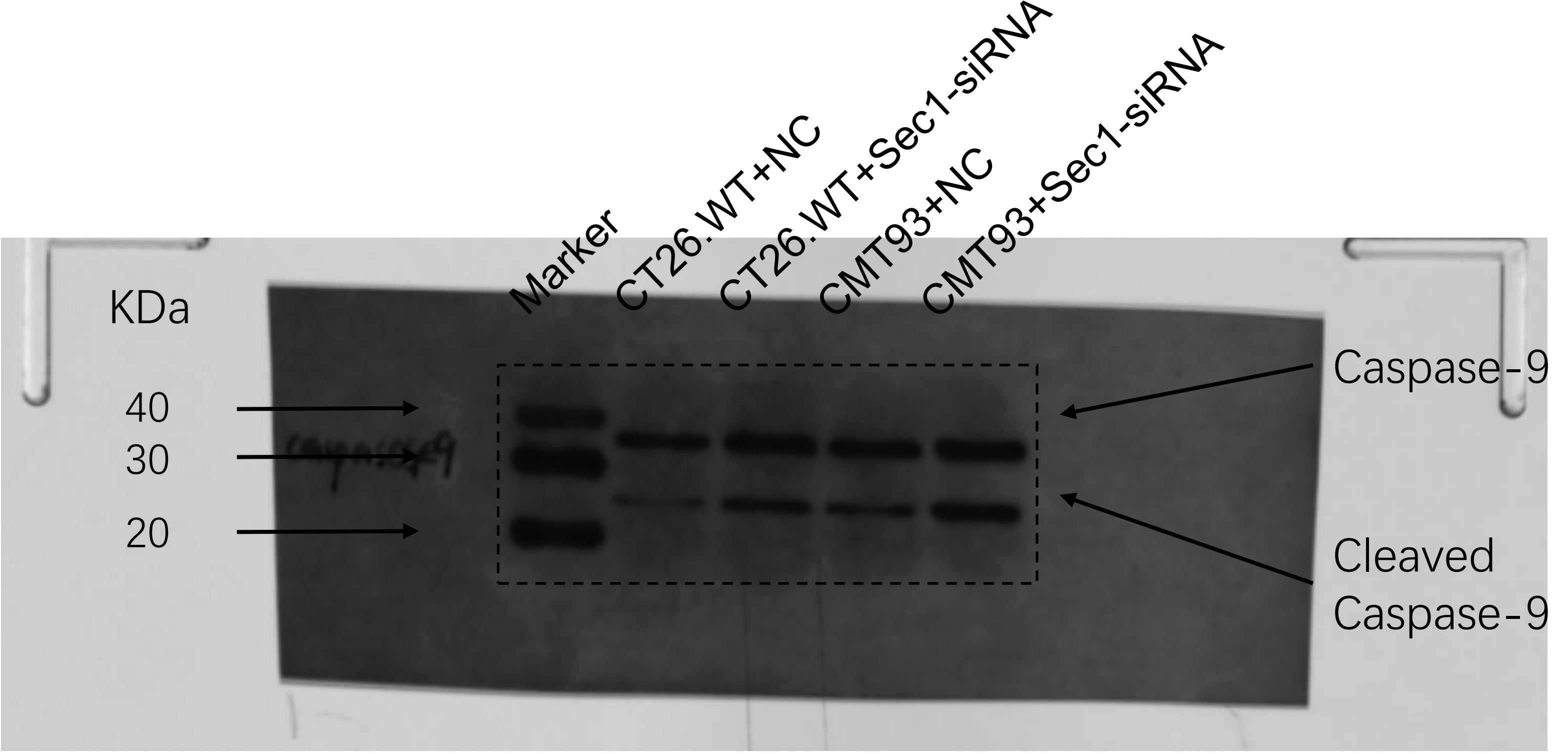

Figure 4E: CASPASE9
